# Synthesis and PI3 Kinase Inhibition Activity of a Wortmannin-Leucine Derivative

**DOI:** 10.3390/molecules23071791

**Published:** 2018-07-20

**Authors:** William Cantrell, Yue Huang, Antonio A. Menchaca, George Kulik, Mark E. Welker

**Affiliations:** 1 Medicinal and Process Chemistry, Pharmaceutical Development and Biomaterials Department, Chemistry and Chemical Engineering Division, Southwest Research Institute, P.O. Drawer 28510, San Antonio, TX 78228, USA; william.cantrell@swri.org (W.C.); menchaca@swri.org (A.A.M.); 2 Department of Cancer Biology and Comprehensive Cancer Center, Wake Forest School of Medicine, Medical Center Blvd., Winston-Salem, NC 27157, USA; yhuang@wakehealth.edu; 3 Life Sciences Program, College of Science, Alfaisal University, Riyadh 11533, Saudi Arabia; 4 Department of Chemistry, Wake Forest University, Winston-Salem, NC 27109, USA

**Keywords:** wortmanin synthesis, PI3K inhibitor, prostate cancer

## Abstract

Wortmannin is a potent covalent inhibitor of PI3K that shows substantial in vivo toxicity and thus is unsuitable for systemic therapeutic applications. One possible approach to minimize systemic toxicity is to generate a latent wortmannin pro-drug that will be selectively activated in target tissues. To test this approach, a wortmannin derivative with a leucine linker attached to C20 has been synthesized and tested for inhibition of PI3K activity in prostate cancer cells. Analysis of PI3K pathway inhibition by Wormannin-Leu (Wn-L) and intact Wortmannin (Wn) showed that attachment of Leu at C-20 decreased potency of PI3K pathway inhibition 10-fold compared to intact wortmannin, yet exceeded the potency of a competitive PI3K inhibitor LY294002.

## 1. Introduction

**Scheme 1:**
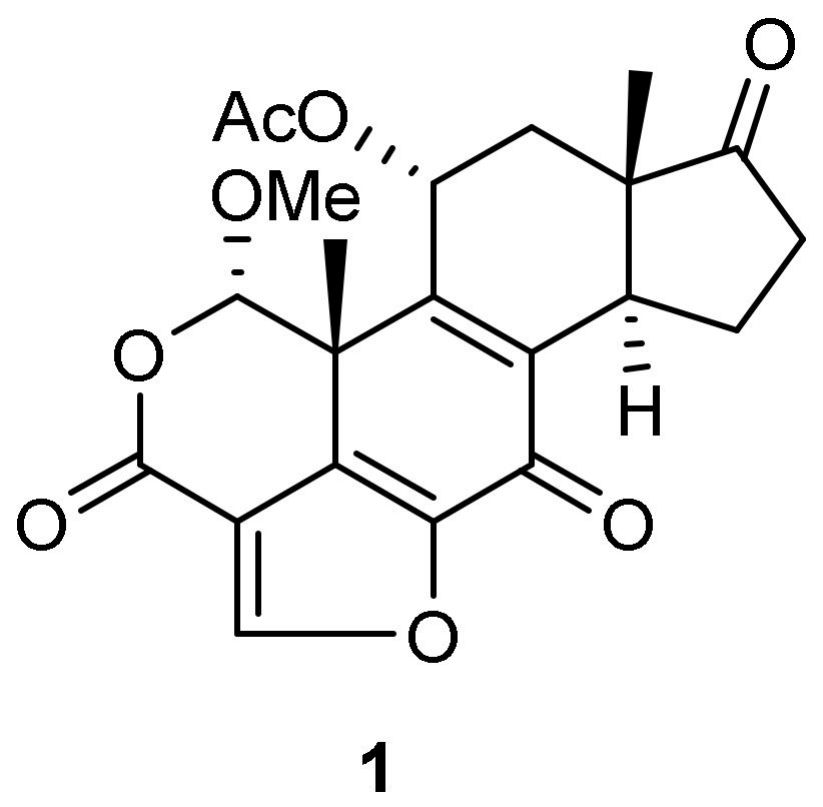
Wortmannin structure.

A significant proportion of advanced prostate cancers are characterized by increased PI3K signaling, however no survival benefits from PI3K inhibitors tested in clinical trials have been reported [1,2,3,4,5,6,7]. One potential strategy to improve therapeutic efficacy is to generate prostate-selective PI3K inhibitor prodrugs by attaching a peptide that prevents entry of the PI3K inhibitor into cells. To test this concept, a prodrug of quercetin analogue LY294002 has been generated by attaching a peptide Mu-LEHSSKLQL, in which the internal sequence HSSKLQ is a substrate for the prostate-specific antigen (PSA) protease. This produced a latent PI3K inhibitor prodrug Mu-LEHSSKLQL-LY294002 which activation is dependent on PSA cleavage. Once activated, Mu-LEHSSKLQL-LY294002 can specifically inhibit PI3K in PSA-secreting prostate cancer cells and induce apoptosis with a potency comparable to that of the original LY294002 compound [8]. However, for effective inhibition of PI3K in vivo, generating prostate-selective PI3K inhibitors with lower IC50 would be beneficial. To generate a more potent prostate-selective PI3K inhibitor, we tested the feasibility of replacing LY294002 (IC50 ~1.4 mcM) with Wortmannin (**1**) (IC50 4.7 nM) (Scheme 1).

Because PSA is predicted to cleave Mu-LEHSSKLQL peptide after glutamine (Q), it leaves leucine (Leu) attached to the base inhibitor that potentially can diminish inhibitor potency. Since earlier publications demonstrated that various moieties can be attached to wortmannin via C-20 [9,10] we tested the effect of Leu attached at this position on the compound potency and we report the results of those studies here.

## 2. Results and Discussion

### 2.1. Chemistry

**Scheme 2:**
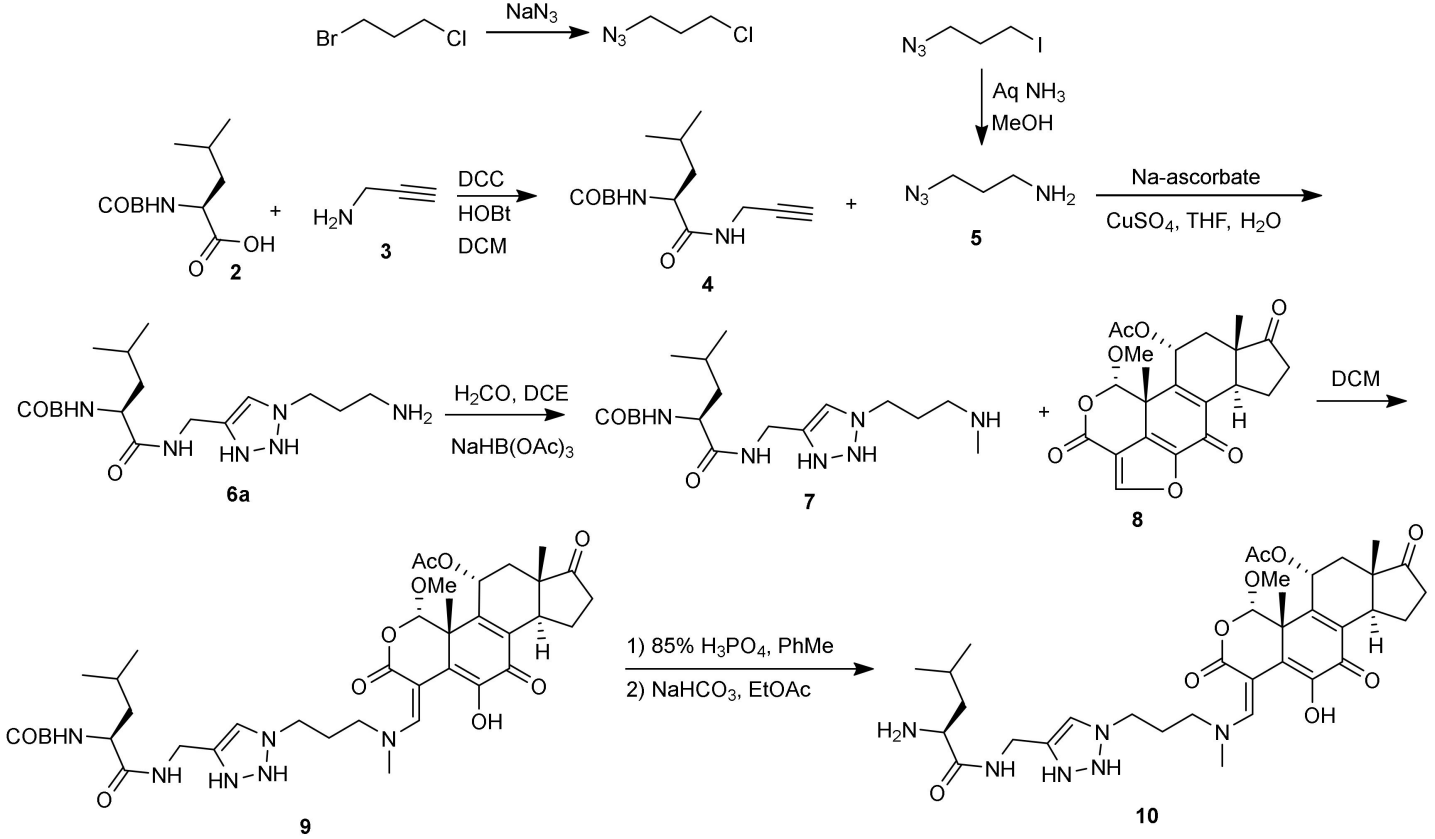
Synthesis of Wortmannin–Leucine compound.

The synthesis of compound **10** (Scheme 2) was accomplished by the reaction of Boc-protected leucine (**2**) with propargylamine (**3**) under standard peptide coupling conditions (diisopropylcarbodiimide (DIC), hydroxybenzotriazole (HOBt)) to give the propargyl amide (**4**) in 90% yield. Azido amine compound (**5**) was prepared as described previously [11]. The click reaction between **4** and **5** produced compound **6** as a 2.3:1 mixture of regioisomers, which were purified by SCX2 resin in 64% yield. We presume based on literature precedent [12], that the trans isomer (**6**) shown was the major isomer due to steric interactions in the cycloaddition. For simplicity this scheme just depicts this isomer. Compound **6** was methylated at the terminal amino group via reductive amination using formalin and sodium triacetoxyborohydride (STAB-H) to form compound **7** in low yield (14%) after extensive purification. The reaction was not selective and a mixture of mono and dialkylated products, along with unreacted starting material (**6**) were present. As this was a proof of concept project, we decided not to optimize any one synthetic step unless the inhibition activity at the end warranted it later. We did not add the primary amine (**6**) directly to wortmannin at C-20 since other primary amines were known to add there reversibly [10]. Compound **7** was coupled with wortmannin (**8**) and the product (**9**) was purified by silica gel chromatography in 38% yield. The Boc protecting group was removed (phosphoric acid, toluene) to form compound (**10**).

### 2.2. Biological Activity

To assess the biological activity of new PI3K inhibitor compounds, the experiments were conducted in C4-2 prostate cancer cells that represent castration-resistant metastatic prostate cancer. C4-2 cells are characterized by constitutive activation of PI3K pathway due to the loss of expression of PI3 phosphatase PTEN. A protein kinase PKB/AKT is among best characterized downstream effectors of PI3K pathway. Activation of PI3K leads to accumulation of PI3P in the plasma membrane. PI3P binds to PH domains of AKT and PDK kinases and recruits AKT and PDK kinases to plasma membrane. This in turn leads to phosphorylation of AKT at T308 by PDK1, and to phosphorylation at S473 by rapamycin-insensitive mTOR:Rictor:Gβ complex. Thus, phosphorylation of AKT at S473 and T308 faithfully reflect activation of PI3K pathway in most cell lines and routinely used to monitor the PI3K activity [13].

**Figure 1:**
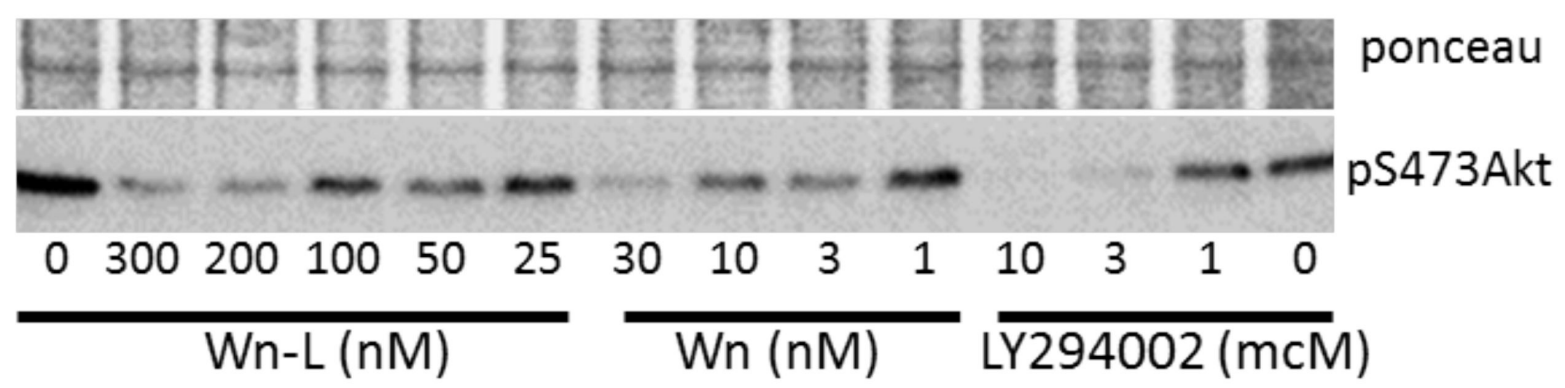
S473AKT phosphorylation in C4-2 prostate cancer cells. C4-2 cells were treated with Wn-L (**10**) (300, 200, 100, 50, 25nM), Wn (**1**) (30, 10, 3, 1 nM) or LY294002 (10, 3, 1, 0 µM) as indicated for 1 h, and analyzed for S473AKT phosphorylation. Region of the membrane corresponding to 60–70 kDa stained with Ponceau S is shown as control of equal loading.

Analyses of phosphorylation of S473AKT in C4-2 cells have been used in this study to assess the PI3K inhibition by Wn-L. Figure 1 shows representative western blots that illustrate inhibition of S473AKT phosphorylation by Wn (**1**) and by Wn-L (**10**) which contains the leucine linker.

Comparison of PI3K pathway inhibition by Wormannin–Leu (Wn-L) (**10**) and intact Wortmannin (Wn) (**1**) showed that attachment of Leu at C-20 decreased potency of PI3K pathway inhibition by 10 fold since comparable inhibition of Akt phosphorylation at S473 was observed in C42 cells treated with 100 nM of Wn-L and 10 nM of Wn (Figure 1). Future studies of the effects of Leu attached at various positions of Wn are needed to determine which Wn–L derivative compound will retain the inhibitory potency of intact wortmannin.

Quantitation of Western blots by image J software showed that respective IC50′s for Wn and Wn–L were 100- and 10-fold lower comparing to the well characterized PI3K inhibitor ZSTK474. Thus, 50% inhibition of S473AKT phosphorylation was observed at lower concentrations of Wn and Wn–L than 50% inhibition by ZSTK474. Comparison of Wn–L and Wn, showed that inhibition of S473AKT phosphorylation by compound Wn–L at 300 nM was comparable to inhibition by Wn at 30 nM; whereas inhibition by Wn–L at 100 nM was comparable to inhibition by Wn at 10 nM. Thus, Wn–L is a 10-fold less potent PI3K inhibitor comparing to Wn.

## 3. Materials and Methods

### 3.1. General Information

Unless otherwise noted, solvents and reagents were used without purification. 1,2-dichloroethylene (DCE) was dried over 4 Å molecular sieves for 48 h prior to use. Volatile solvents were removed under reduced pressure using a Buchi rotary evaporator. Infrared (IR) spectra were obtained using a Nicolet iS550 FT IR spectrophotometer using a diamond crystal attenuated total reflection (ATR) accessory and reported as wave numbers. Melting points were determined using differential scanning calorimetry (DSC) on a TA Instruments Differential Scanning Calorimeter Model Q100. Thin layer chromatography (TLC) was performed on glass-backed precoated silica gel plates (0.25 mm thick with 60 F254) and were visualized using one or both of the following manners: UV light (254 nm) and staining with I_2_ impregnated silica. Flash flash chromatography was performed using the Biotage Isolera One using pre-loaded Silicycle 25g high performance (14–40 μM) columns. ^1^H nuclear magnetic resonance (NMR) spectra were obtained at 400 MHz as indicated as solutions in CDCl_3_ with 0.05% v/v tetramethylsilane (TMS) unless indicated otherwise. ^13^C-NMR were obtained at 100 MHz as shown in the indicated deuterated solvent. Chemical shifts are reported in parts per million (ppm, δ), and referenced to TMS, and coupling constants are reported in Hertz (Hz). Spectral splitting patterns are designated as s, singlet; d, doublet; t, triplet; q, quartet; quint, quintuplet; sex, sextet; sept, septuplet; m, multiplet; comp, overlapping multiplets of magnetically nonequivalent protons; br, broad; and app, apparent. Copies of NMR spectra and mass spectra for all new compounds reported here are included in the Supplementary Materials.

### 3.2. Compound Synthesis

#### 3.2.1. *Tert*-Butyl [(2*S*)-1-(Thynylamino)-4-methyl-1-oxopentan-2-yl]carbamate (**4**)

A 100 mL flask was charged with Boc-leucine (**2**, 2.54 g, 10.19 mmol), propargyl amine (**3**, 0.62 g, 11.20 mmol), HOBt hydrate (0.16 g, 1.02 mmol) and dry DCM (20 mL). Diisopropylcarbodiimide (DIC, 1.35 g, 4.92 mmol) was added and the mixture was stirred at ambient temperature for 2 h. Analysis by LCMS showed consumption of Boc-leucine. The reaction mixture was filtered and the filtrate was concentrated. EtOAc (30 mL) was added and the solution was washed with 0.5 M HCl (15 mL), H_2_O (15 mL), and saturated NaHCO_3_ (15 mL). The organic portion was dried (Na_2_SO_4_) and concentrated. The residue was suspended in hexanes (30 mL) and the mixture was filtered. The filtrate was concentrated to give **4** as a white solid (2.45 g, 9.13 mmol, 90%). ^1^H-NMR (CDCl_3_) δ 6.69 (br s, 1H), 4.99 (br d, 1H, *J* = 8), 4.15 (br s, 1H), 4.04 (dd, 2H, *J* = 5, 2), 2.22 (t, 1H, *J* = 3), 1.70–1.40 (m, 3H), 1.45 (s, 9H), 0.94 (t, 6H, *J* = 6), 1.00–0.85 (m, 1H). ^13^C-NMR (CDCl_3_) 172.8, 155.9, 79.9, 79.4, 71.4, 71.4, 52.8, 41.4, 29.0, 28.3, 24.6, 23.4, 22.9, 21.9 ppm. MS *m*/*z* [M + Na]^+^ = 291.2.

#### 3.2.2. Boc-Leu-NHCH_2_-triazole-(CH_2_)_3_NH_2_ (**6a**, **b**)

A 40 mL vial was charged with 3-azidopropan-1-amine (**5**, 0.509 g, 5.08 mmol) and a solution of Boc-Leu-NHCCH (**4**, 1.43 g, 5.34 mmol) in 1/1 THF/H_2_0 (2 mL) was added. A solution of CuSO_4_ H_2_O (0.063 g, 0.25 mmol) in 1/1 THF/H_2_O (2 mL) was added followed by a solution of sodium ascorbate (0.101 g, 0.51 mmol) in 1/1 THF/H_2_O (2 mL). The mixture was stirred at room temperature for 30 min. Analysis by LCMS showed complete conversion. Solid NH_4_Cl (1.1 g) and 30% NH_4_OH (0.76 g) were added, and the mixture was extracted with EtOAc (2 × 25 mL). The combined organic portions were dried (Na_2_SO_4_) and concentrated. The crude product was dissolved in DCM (5 mL) and the solution was charged to an SCX column (12 g). The column was eluted with a gradient mixture, 0 to 65%, 0.5M NH_3_ in MeOH/DCM, over 15 column volumes. The pure fractions were combined and the impure fractions were repurified (2×) to give **6** as a glassy solid (1.19 g, 3.23 mmol, 64%, mixture of regioisomers). ^1^H-NMR (CDCl_3_) δ 7.57 (s, 1H), 7.03 (br t, 1H), 5.00 (d, 1H, *J* = 8), 4.51 (t, 2H, *J* = 6), 4.44 (t, 2H, *J* = 7), 4.12 (br s, 1H), 2.72 (t, 2H, *J* = 6), 2.02 (t, 2H, *J* = 6), 1.73–1.60 (m, 9H), 1.42 (s, 9H), 0.92 (dd, 6H, *J* = 6, 2). ^13^C-NMR (CDCl_3_) 173.1, 155.8, 144.7, 122.6, 80.0, 53.2, 47.6, 41.5, 38.2, 34.9, 32.3, 28.3, 24.7, 23.1, 21.8 ppm. MS *m*/*z* [M + H]^+^ = 369.3.

#### 3.2.3. Boc-Leu-NHCH_2_-triazole-(CH_2_)_3_NHMe (**7**)

A 250 mL flask was charged with **6** (1.47 g, 3.99 mmol) and 1,2-DCE (80 mL), Sodium triacetoxyborohydride (STAB-H, 0.85 g, 3.99 mmol) was added as a solid in one portion. Formaldehyde (37 wt% aqueous solution, 0.32 g, 3.99 mmol) was added dropwise over 5 min and the mixture was stirred at ambient temperature for 24 min. Analysis by LCMS showed some compound **6** remaining, so more STAB-H (0.85 g, 3.99 mmol) was added. The mixture was stirred for 16 hours at ambient temperature. Saturated NaHCO_3_ (950 mL) was slowly added to the reaction mixture (off-gassing). The top aqueous layer was extracted with DCM (2 × 30 mL) and the combined organic portions were combined, dried (Na_2_SO_4_) and concentrated to give the crude product (1.68 g). Purification by normal phase chromatography (40 g silica, gradient, 0 to 33% 0.5M NH_3_/MeOH in DCM over 11 column volumes) gave mostly pure **7** (0.22 g, 14%). This material was combined with other lots of **7** from additional reactions for further purification. Chromatographic purification was performed twice (260 mg of once purified **7**, 4 g silica, gradient, 0 to 33% 0.5M NH_3_/MeOH in DCM over 11 column volumes)), produced pure **7** (0.143 g, 55%). ^1^H NMR (CDCl_3_) δ 7.57 (d, 1H, *J* = 2), 7.02 (br d, 1H, *J* = 3), 5.00 (br d, 1H, *J* = 8), 4.51 (t, 2H, *J* = 6), 4.43 (q, 2H, *J*= 7), 4.13 (br s, 1H), 2.71 (regioisomer A, t, 1H, *J* = 7), 2.58 (regioisomer B, t, 1H, *J* = 7), 2.41 (s, 2H), 2.09-1.98 (m, 2H), 1.74–1.58 (m, 6H), 1.42 (s, 9H), 0.92 (dd, 6H, *J* = 6, 2). ^13^C-NMR (CDCl_3_) 173.2, 155.8, 144.8, 122.6, 79.9, 53.2 48.2, 48.1, 47.7, 41.6, 38.5, 36.0, 35.0, 33.1, 29.8, 28.3, 24.7, 23.1, 21.8 ppm. MS *m*/*z* [M + H]^+^ = 383.3.

#### 3.2.4. Boc-Leu-NHCH_2_-triazole-(CH_2_)_3_NHMe-wortmannin (**9**)

A 20 mL vial was charged with **7** (mixture of regioisomers, 0.032 g, 0.084 mmol) and DCM (1 mL). A solution of wortmannin (**1**) (0.025 g, 0.058 mmol) in DCM (1 mL) was added and the mixture was stirred at ambient temperature for 1.5 h. The reaction mixture was concentrated and purified by normal phase silica (1/1, EtOAc/DCM, then 100% EtOAc, then 100% acetone) to give the product (**9**) as an orange solid (0.0183 g, 0.022 mmol, 38%). ^1^H-NMR (CDCl_3_) δ 9.69 (br t, 1H, *J* = 7), 8.48 (d, 1H, *J* = 14), 8.07 (s, 1H), 7.68 (s, 1H), 7.61 (s, 1H), 7.49 (s, 2H), 7.00 (br s, 1H), 6.09 (br t, 1H, J = 7), 5.99 (dd, 1H, *J* = 8, 3), 5.25 (br d, 1H), 4.75-4.30 (m, 12H), 3.60-2.80 (m, 10H), 3.27 (s, 1.5H), 3.24 (s, 1.5H), 2.65–1.45 (m, 34H). MS *m*/*z* [M + H]^+^ = 811.4, [M + Na]^+^ = 833.5.

#### 3.2.5. Leu-NHCH_2_-triazole-(CH_2_)_3_NHMe-wortmannin (**10**)

A 100 mL flask was charged with **9** (0.0183 g, 0.022 mmol) and toluene (2 mL). Phosphoric acid (85%, 0.1 mL) was added and the mixture was stirred at ambient temperature for 23 min. Water (1 mL) and EtOAc (2 mL) were added. Solid NaHCO_3_ was added until the off-gassing stopped (pH = 8). The mixture was extracted with EtOAc (3 × 2 mL). Solid NaCl was added. The mixture was extracted with more EtOAc (3 × 2 mL). The combined organic portions were dried (Na_2_SO_4_) and concentrated to give an orange solid (0.0150 g, 0.020 mmol 93%). ^1^H-NMR (CDCl_3_) δ (see appendix). MS *m*/*z* [M + H]^+^ = 711.3, [M + Na]^+^ = 733.3.

## 4. Conclusions

In conclusion, we conducted the synthesis and pilot characterization of a new wortmannin derivative PI3K inhibitor Wn–L with increased potency compared to the well-characterized PI3K inhibitor ZSTK474. Future studies of the effects of Leu attached at various positions of Wn are needed to determine which Wn–L derivative compound will retain the inhibitory potency of intact wortmannin.

## Supplementary Materials

Supplementary Materials are available online.

## References

[1] Chang L., Graham P.H., Ni J., Hao J., Bucci J., Cozzi P.J., Li Y. (2015). Targeting PI3K/Akt/mTOR signaling pathway in the treatment of prostate cancer radioresistance. Crit. Rev. Oncol. Hematol..

[2] Sarker D., Reid A.H.M., Yap T.A., de Bono J.S. (2009). Targeting the PI3K/AKT Pathway for the Treatment of Prostate Cancer. Clin. Cancer Res..

[3] Toren P., Zoubeidi A. (2014). Targeting the PI3K/Akt pathway in prostate cancer: Challenges and opportunitie. Int. J. Oncol..

[4] Uzoh C.C., Perks C.M., Bahl A., Holly J.M.P., Sugiono M., Persad R.A. (2009). PTEN-mediated pathways and their association with treatment-resistant prostate cancer. BJU Int..

[5] Taylor B.S., Schultz N., Hieronymus H., Gopalan A., Xiao Y., Carver B.S., Arora V.K., Kaushik P., Cerami E., Reva B. (2010). Gerald, Integrative Genomic Profiling of Human Prostate Cancer. Cancer Cell..

[6] Ma X., Hu Y. (2013). Targeting PI3K/Akt/mTOR Cascade: The Medicinal Potential, Updated Research Highlights and Challenges Ahead. Curr. Med. Chem..

[7] Asif K.Q., Aashiq H., Abid H., Yasrib Q., Rabiya M., Mudassier A., Rauf A.N., Javeed A.B., Shashank K.S., Mohmmad A.Z. (2013). Recent Development in Targeting PI3K-Akt-mTOR Signaling for Anticancer Therapeutic Strategies. Anti-cancer Agent. Med. Chem..

[8] Baiz D., Pinder T.A., Hassan S., Karpova Y., Salsbury F., Welker M.E., Kulik G. (2012). Synthesis and Characterization of a Novel Prostate Cancer-Targeted Phosphatidylinositol-3-kinase Inhibitor Prodrug. J. Med. Chem..

[9] Wipf P., Minion D.J., Halter R.J., Berggren M.I., Ho C.B., Chiang G.G., Kirkpatrick L., Abraham R., Powis G. (2004). Synthesis and biological evaluation of synthetic viridins derived from C(20)-heteroalkylation of the steroidal PI-3-kinase inhibitor wortmannin. Org. Biomol. Chem..

[10] Yuan H., Luo J., Weissleder R., Cantley L., Josephson L. (2006). Wortmannin-C20 conjugates generate wortmannin. J. Med. Chem..

[11] Lebreton L., Jost E., Carboni B., Annat J., Vaultier M., Dutartre P., Renaut P. (1999). Structure−Immunosuppressive Activity Relationships of New Analogues of 15-Deoxyspergualin. 2. Structural Modifications of the Spermidine Moiety. J. Med. Chem..

[12] Singh M.S., Chowdhury S., Koley S. (2016). Advances of azide-alkyne cycloaddition-click chemistry over the recent decade. Tetrahedron.

[13] Bayascas J.R., Alessi D.R. (2005). Regulation of Akt/PKB Ser473 Phosphorylation. Mol. Cell.

