# Supplementary material for: Synthesis and PI3 Kinase Inhibition Activity of a Wortmannin-Leucine Derivative
Source: Molecules. 2018 Jul 20;23(7):1791. doi: 10.3390/molecules23071791 (PMC6100554; doi:10.3390/molecules23071791)

13-0203-011-12-31
W. Carter 05/28/15

13-0203-011-12-2
crude product

exp1 Proton

```
      SAMPLE                   SPECIAL
date    May 28 2015  temp          24.0
solvent        cdcl3  gain      not used
file /home/pharmd/~   spin            20
vnmrsys/data/auto_~   hst          0.008
2014.11.19/00699.f~   pw90        12.750
                  id  alfa         6.600
   ACQUISITION             FLAGS
sw            6399.0  il               n
at             2.049  in               n
np             26218  dp               y
fb              4000  hs              nn
bs                32        PROCESSING
ss                 2  fn           65536
d1             1.000         DISPLAY
nt                16  sp           286.1
ct                16  wp          2651.7
   TRANSMITTER        rfl          784.6
tn                H1  rfp              0
sfrq         399.949  rp           -91.0
tof            399.9  lp           -72.7
tpwr              50          PLOT
pw             6.375  wc             250
   DECOUPLER          sc               0
dn               C13  vs              42
dof                0  th               3
dm               nnn  ai   cdc  ph
dmm                c
dpwr              40
dmf            17100
```

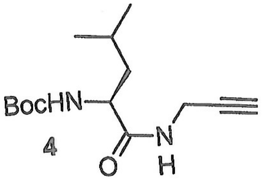

4

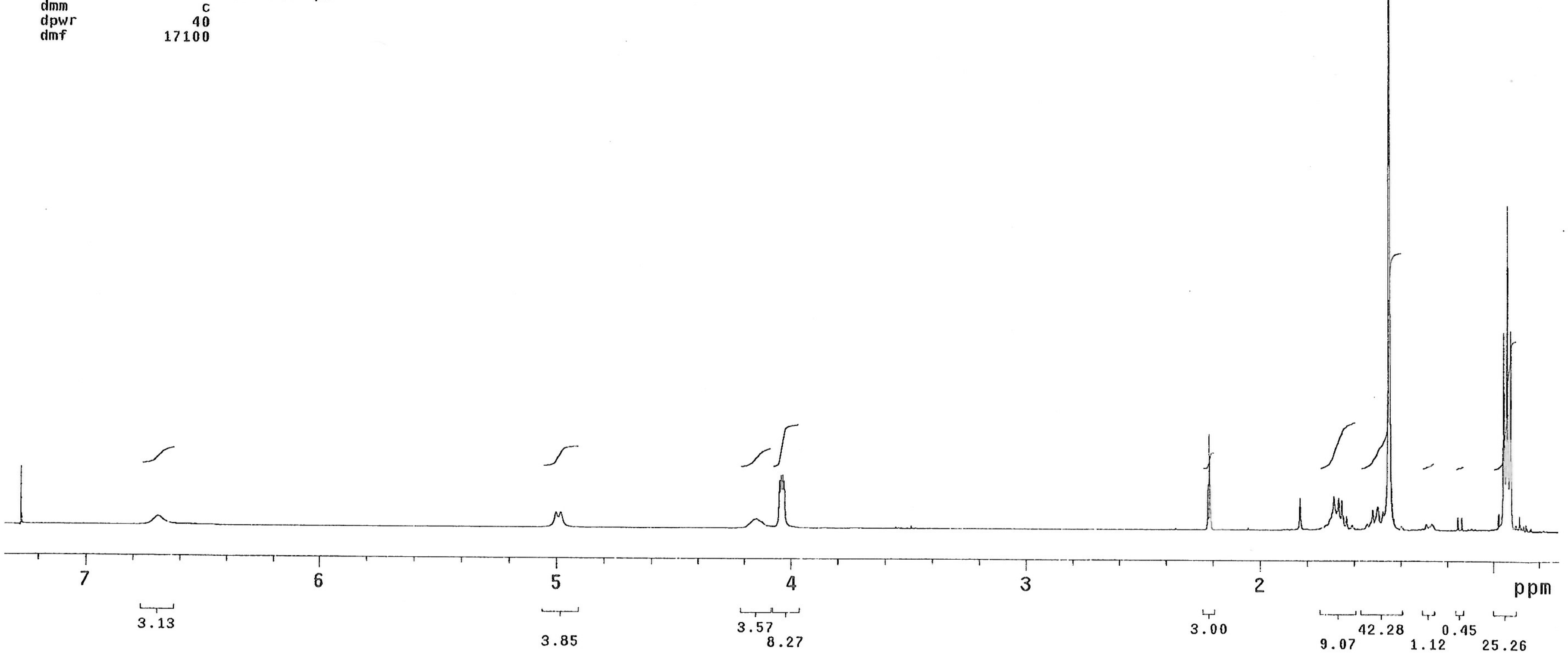

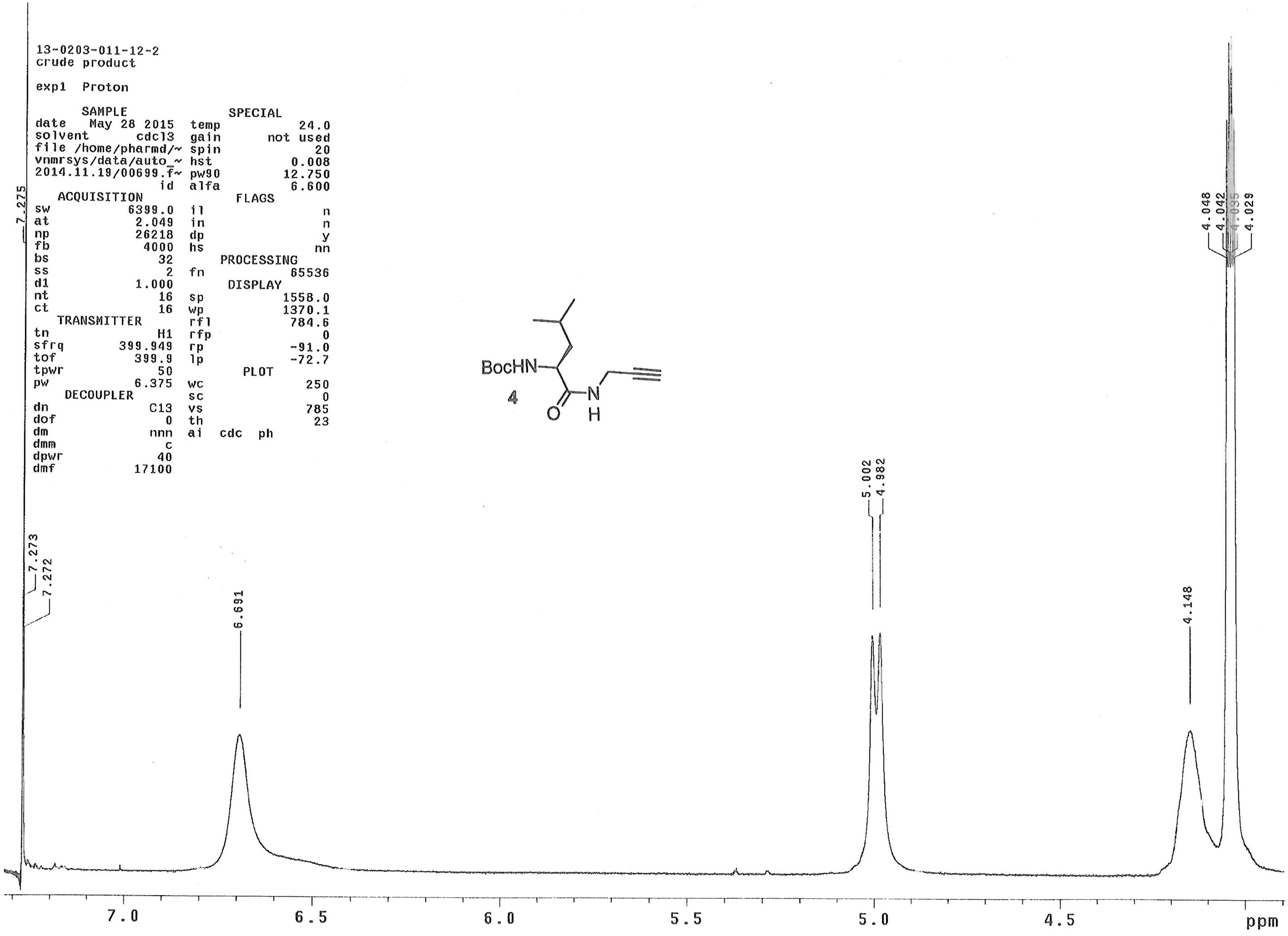
13-0203-011-12-2
crude product
exp1 Proton
SAMPLE
date May 28 2015
solvent cdcl3
file /home/pharmd/~
vnmrsys/data/auto_~
2014.11.19/00699.f~
id
SPECIAL
temp 24.0
gain not used
spin 20
hst 0.008
pw90 12.750
alfa 6.600
ACQUISITION
sw 6399.0
at 2.049
np 26218
fb 4000
bs 32
ss 2
d1 1.000
nt 16
ct 16
FLAGS
il n
in n
dp y
hs nn
PROCESSING
fn 65536
DISPLAY
sp 1558.0
wp 1370.1
rfl 784.6
rfp 0
rp -91.0
lp -72.7
TRANSMITTER
tn H1
sfrq 399.949
tof 399.9
tpwr 50
pw 6.375
PLOT
wc 250
sc 0
vs 785
th 23
ai cdc ph
DECOUPLER
dn C13
dof 0
dm nnn
dmm c
dpwr 40
dmf 17100
BocHN
4
O
N
H
7.275
7.273
7.272
6.691
5.002
4.982
4.148
4.048
4.042
4.035
4.029
7.0
6.5
6.0
5.5
5.0
4.5
ppm

```
13-0203-011-12-2
crude product

exp1  Proton

     SAMPLE                 SPECIAL
date   May 28 2015  temp          24.0
solvent      cdcl3  gain      not used
file /home/pharmd/~ spin            20
vnmrsys/data/auto_~ hst          0.008
2014.11.19/00699.f~ pw90        12.750
                id  alfa         6.600
   ACQUISITION               FLAGS
sw          6399.0  il               n
at           2.049  in               n
np           26218  dp               y
fb            4000  hs              nn
bs              32        PROCESSING
ss               2  fn           65536
d1           1.000          DISPLAY
nt              16  sp           321.2
ct              16  wp           589.9
   TRANSMITTER      rfl          784.6
tn              H1  rfp              0
sfrq       399.949  rp           -91.0
tof          399.9  lp           -72.7
tpwr            50          PLOT
pw           6.375  wc             250
    DECOUPLER       sc               0
dn             C13  vs              42
dof              0  th               6
dm             nnn  ai   cdc   ph
dmm              c
dpwr            40
dmf          17100
```

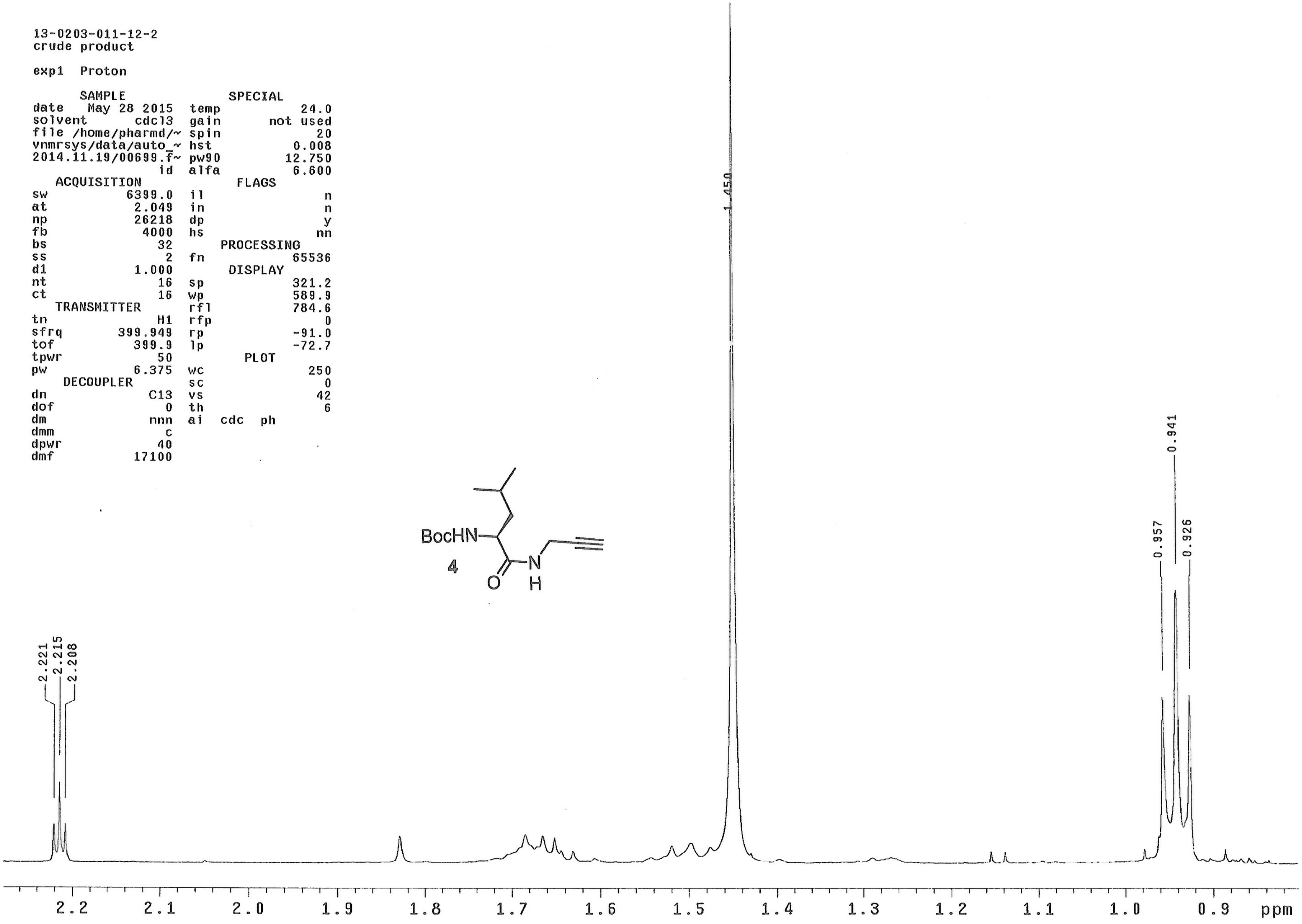

13-0203-011-01-37B
W. Carter 08/23/13

13-0203-011-01-36

exp2 Carbon

SAMPLE		SPECIAL	
date	Aug 22 2013	temp	24.0
solvent	cdcl3	gain	30
file	/home/stan/vn~	spin	20
mrsys/data/auto_20~		hst	0.008
13.07.08/s2pul_81.~		pw90	10.300
	fid	alfa	6.600
ACQUISITION		FLAGS	
sw	24125.5	il	n
at	1.300	in	n
np	62750	dp	y
fb	13000	hs	nn
bs	64	PROCESSING	
d1	5.000	lb	0.50
nt	512	fn	not used
ct	512	DISPLAY	
TRANSMITTER		sp	1971.0
tn	C13	wp	15563.6
sfrq	100.541	rfl	1507.1
tof	1028.1	rfp	0
tpwr	53	rp	129.4
pw	3.438	lp	-114.6
DECOUPLER		PLOT	
dn	H1	wc	250
dof	0	sc	0
dm	yyy	vs	16977
dmm	w	th	8
dpwr	33	ai	cdc ph
dmf	9600		

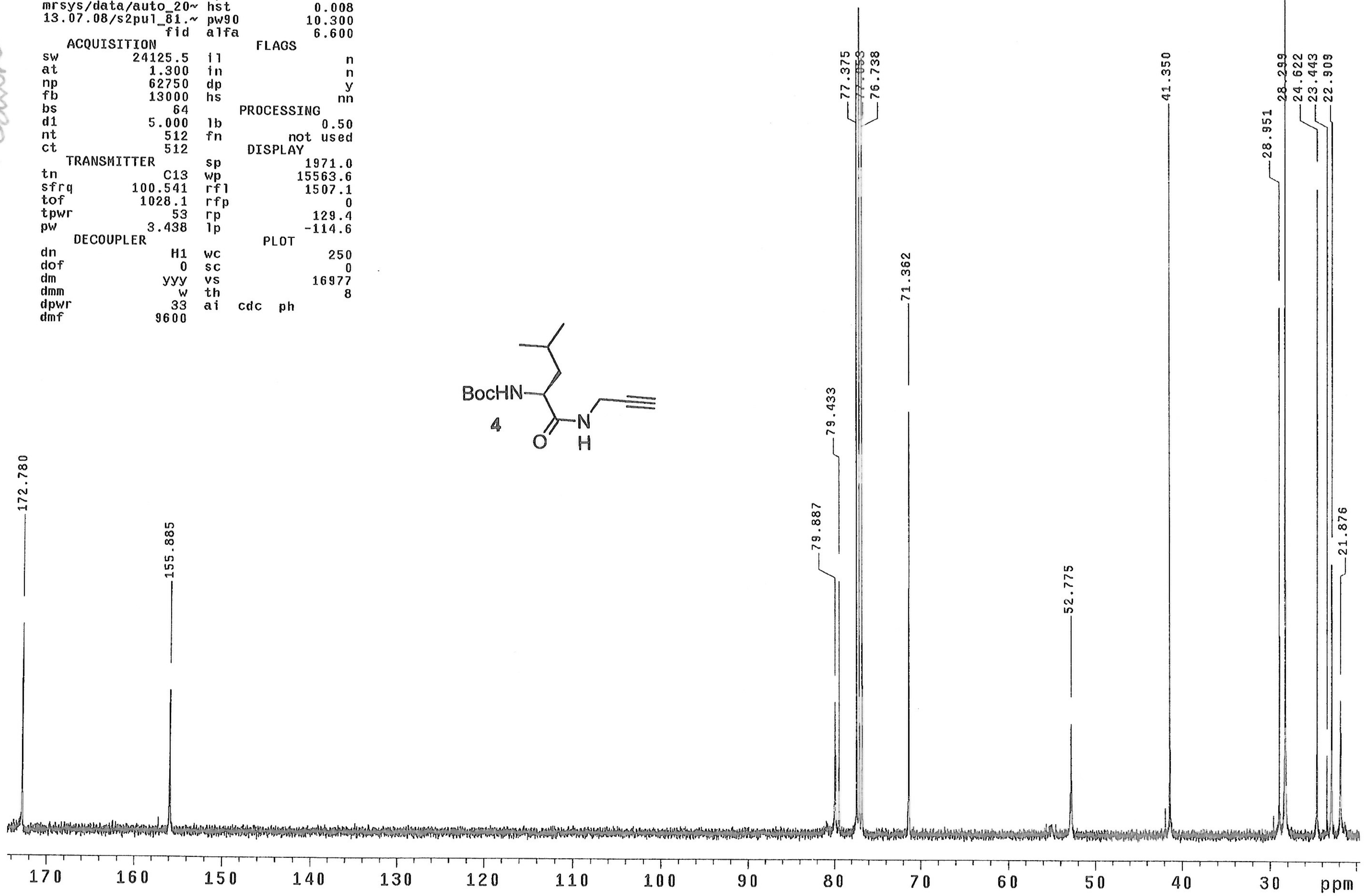

Print of window 80: MS Spectrum
13-0203-011-12-2
crude product

```
=====================================================================
Injection Date  : 5/29/2015 12:18:20 PM          Seq. Line :   1
Sample Name     : 130203011-12-2                  Location : Vial 14
[illegible]. Operator   : Synthesis                              Inj :   1
[illegible]. Instrument : Instrument 1                  Inj Volume : 2 µl
Sequence File   : C:\HPCHEM\1\SEQUENCE\SYN2013.S
Method          : C:\HPCHEM\1\METHODS\GENPOS-S.M
Last changed    : 3/23/2015 2:38:34 PM by Ming
General use pos short run time
```

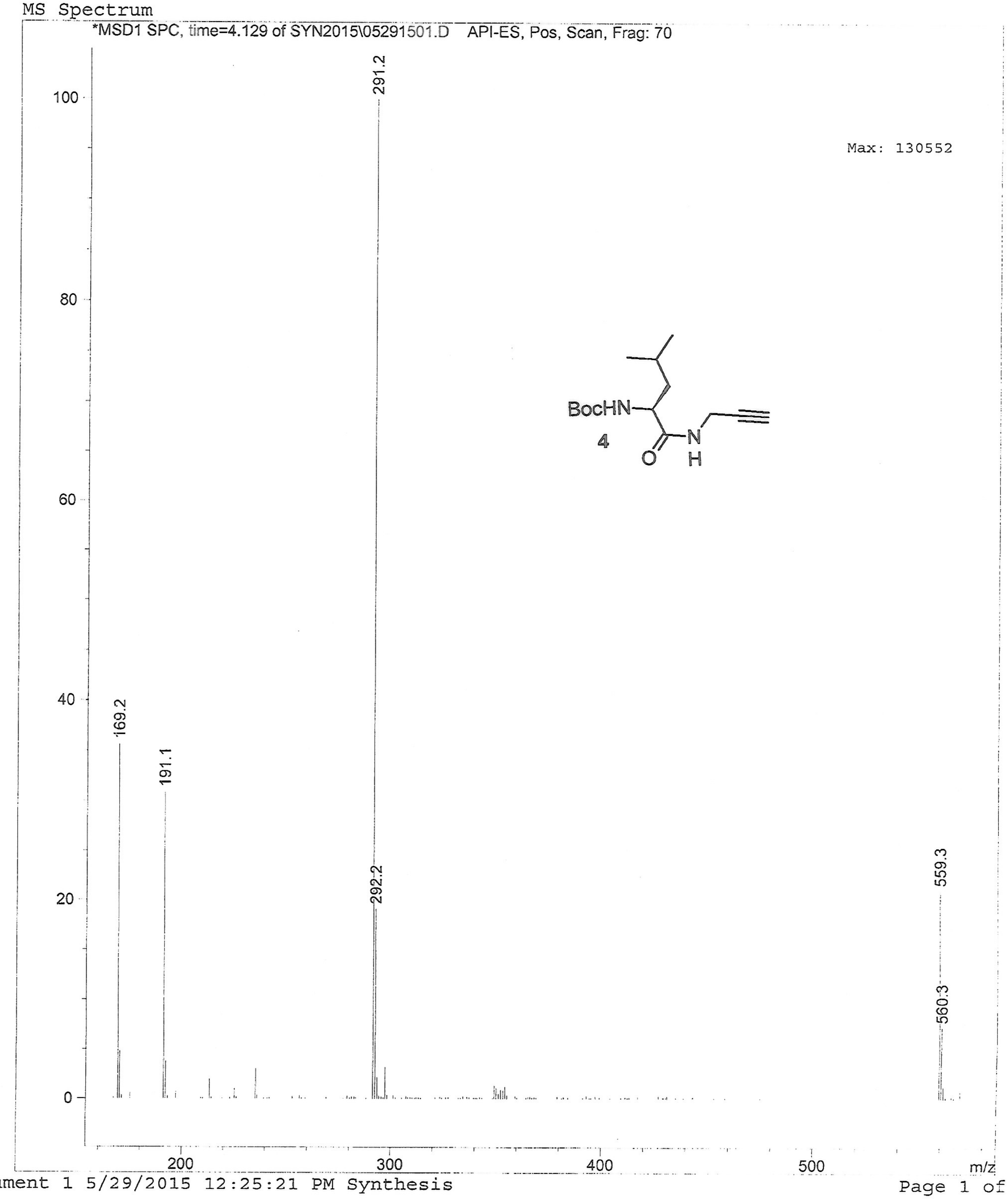

Instrument 1 5/29/2015 12:25:21 PM Synthesis

```
13-0203-011-15-2
Boc-Leu-Click pure

exp1  Proton

      SAMPLE                    SPECIAL
date    Jun 16 2015    temp          24.0
solvent       cdcl3    gain      not used
file /home/pharmd/~    spin            20
vnmrsys/data/auto_~    hst          0.008
2015.06.09/0026.fi~    pw90        12.750
                  d    alfa         6.600
    ACQUISITION                  FLAGS
sw           6399.0    il               n
at            2.049    in               n
np            26218    dp               y
fb             4000    hs              nn
bs               32          PROCESSING
ss                2    fn           65536
d1            1.000            DISPLAY
nt               16    sp           130.1
ct               16    wp          2980.8
    TRANSMITTER        rfl          763.7
tn               H1    rfp              0
sfrq        399.949    rp           -98.2
tof           399.9    lp           -58.1
tpwr             50              PLOT
pw            6.375    wc             250
     DECOUPLER         sc               0
dn              C13    vs             133
dof               0    th               3
dm              nnn    ai    cdc   ph
dmm               c
dpwr             40
dmf           17100
```

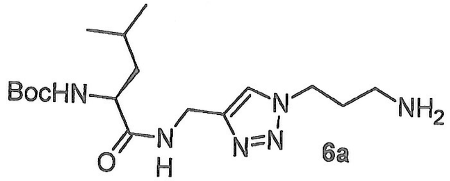

6a

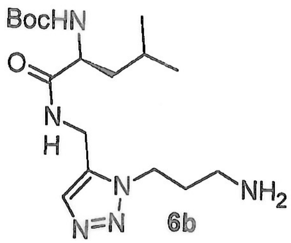

6b

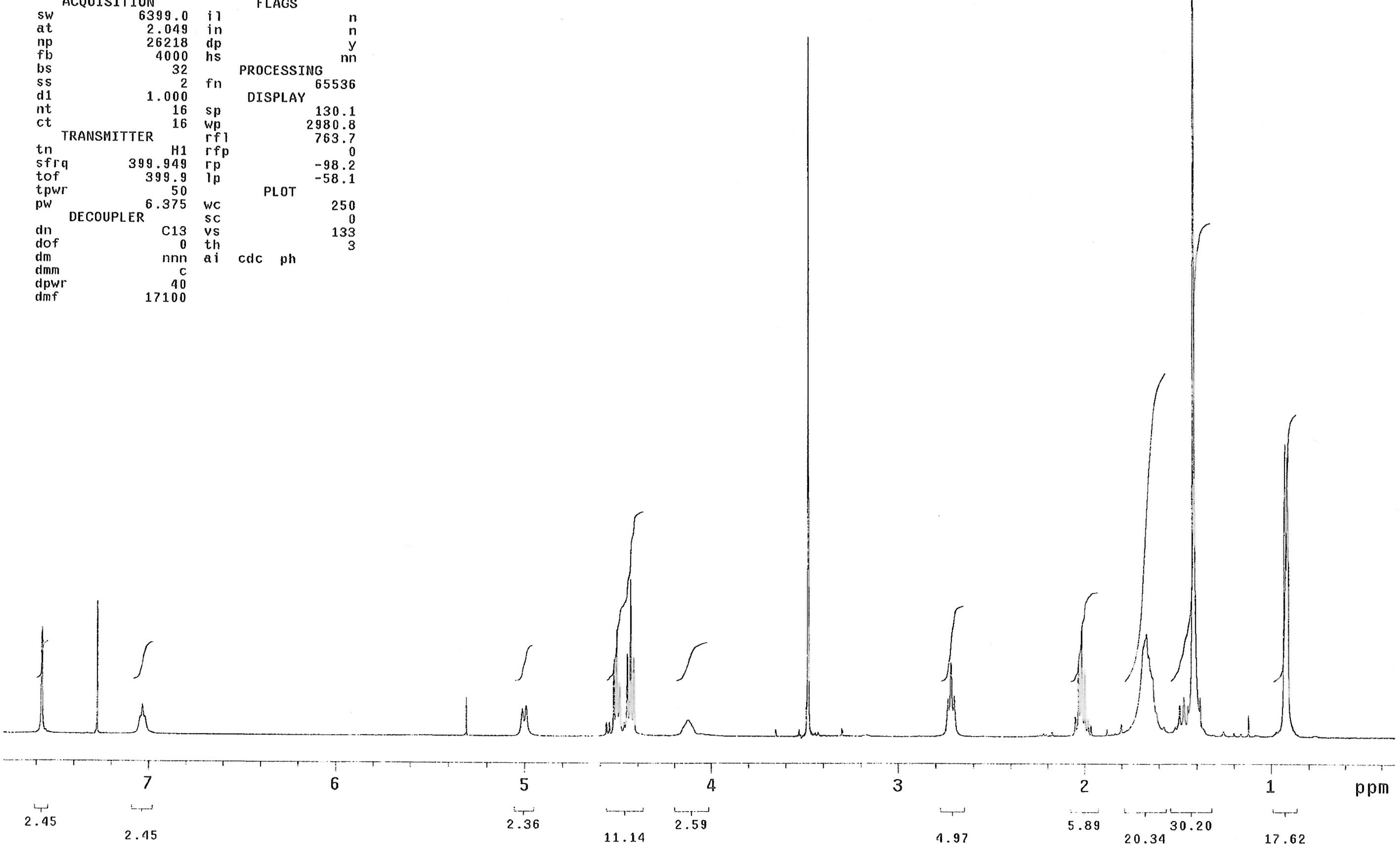

13-0203-011-15-2
Boc-Leu-Click pure

exp1 Proton

SAMPLE		SPECIAL	
date	Jun 16 2015	temp	24.0
solvent	cdcl3	gain	not used
file /home/pharmd/~		spin	20
vnmrsys/data/auto_~		hst	0.008
2015.06.09/0026.fi~		pw90	12.750
	d	alfa	6.600
ACQUISITION		FLAGS	
sw	6399.0	il	n
at	2.049	in	n
np	26218	dp	y
fb	4000	hs	nn
bs	32	PROCESSING	
ss	2	fn	65536
d1	1.000	DISPLAY	
nt	16	sp	2749.4
ct	16	wp	361.3
TRANSMITTER		rfl	763.7
tn	H1	rfp	0
sfrq	399.949	rp	-98.2
tof	399.9	lp	-58.1
tpwr	50	PLOT	
pw	6.375	wc	250
DECOUPLER		sc	0
dn	C13	vs	661
dof	0	th	13
dm	nnn	ai cdc ph	
dmm	c		
dpwr	40		
dmf	17100		

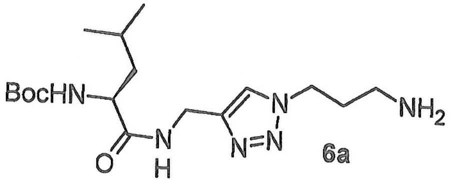

6a

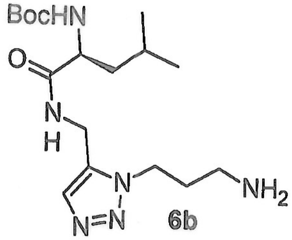

6b

7.573

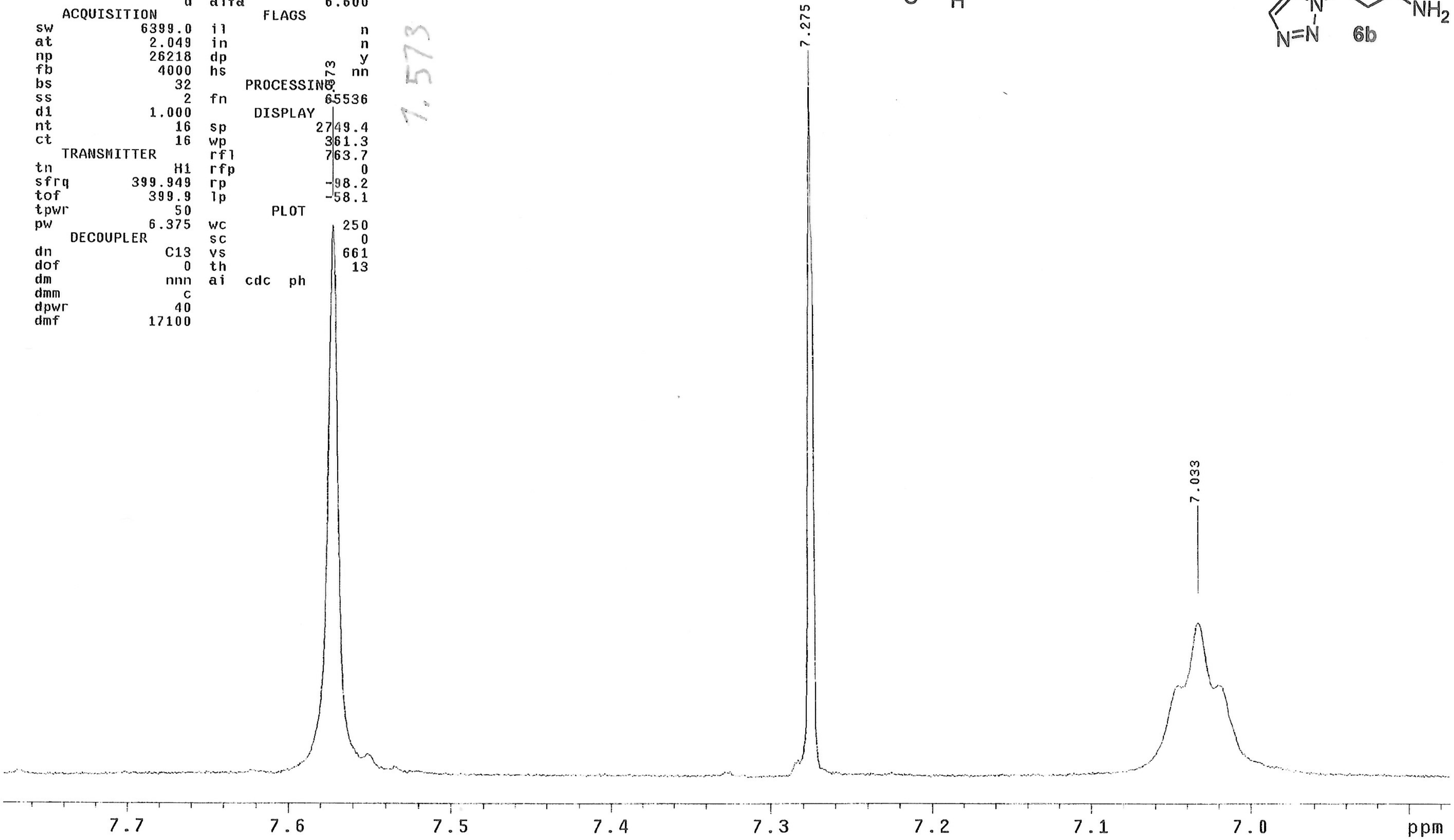

```
13-0203-011-15-2
Boc-Leu-Click pure

exp1  Proton

      SAMPLE                   SPECIAL
date    Jun 16 2015  temp          24.0
solvent       cdcl3  gain      not used
file /home/pharmd/~  spin            20
vnmrsys/data/auto_~  hst          0.008
2015.06.09/0026.fl~  pw90        12.750
                  d  alfa         6.600
   ACQUISITION               FLAGS
sw           6399.0  il               n
at            2.049  in               n
np            26218  dp               y
fb             4000  hs              nn
bs               32        PROCESSING
ss                2  fn           65536
d1            1.000          DISPLAY
nt               16  sp          1530.0
ct               16  wp           629.0
   TRANSMITTER       rfl          763.7
tn               H1  rfp              0
sfrq        399.949  rp           -98.2
tof           399.9  lp           -58.1
tpwr             50           PLOT
pw            6.375  wc             250
    DECOUPLER        sc               0
dn              C13  vs             426
dof               0  th               7
dm              nnn  ai   cdc  ph
dmm               c
dpwr             40
dmf           17100
```

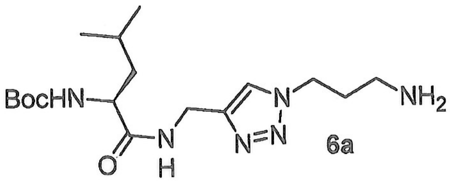

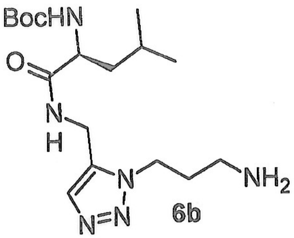

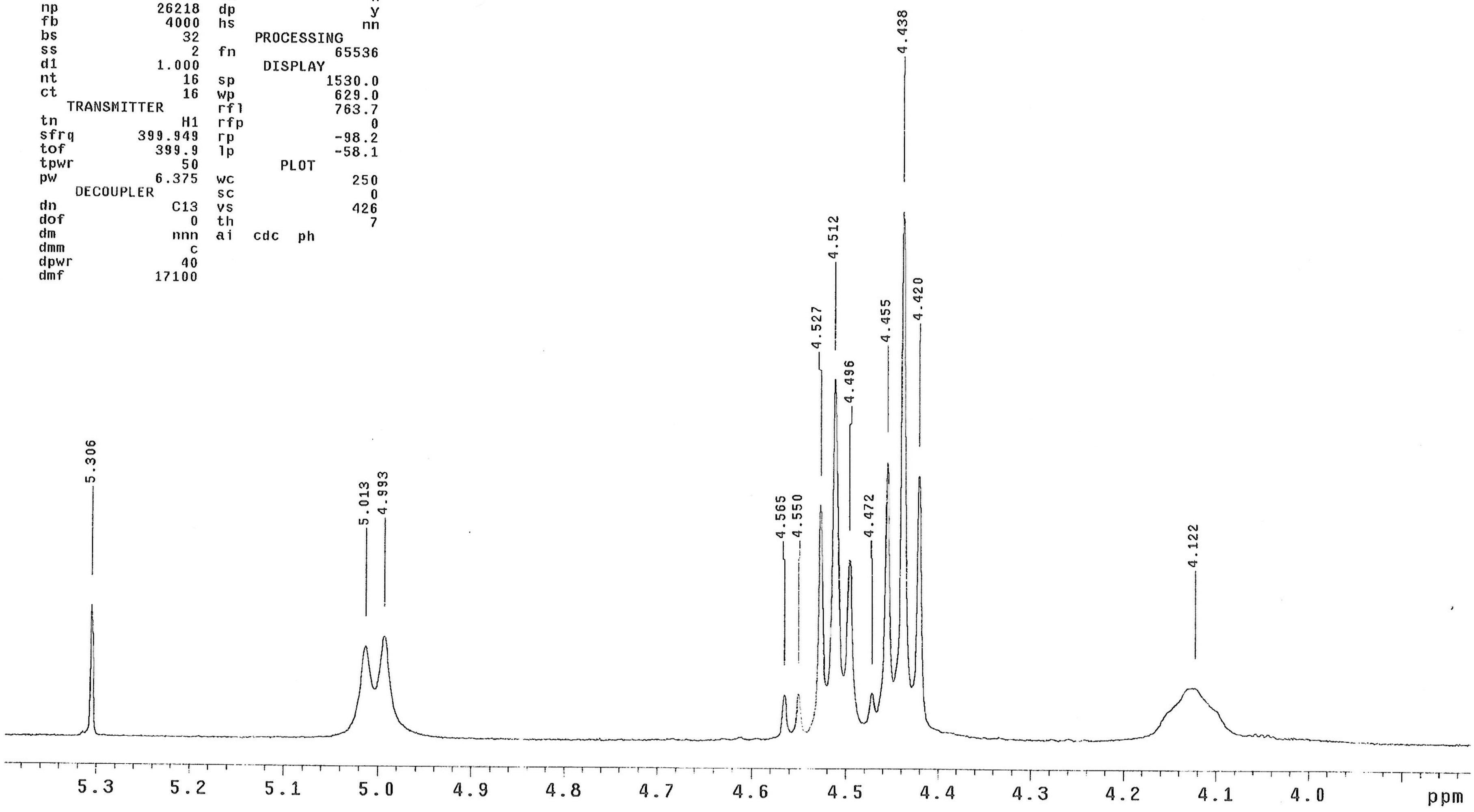

13-0203-011-15-2
Boc-Leu-Click pure

exp1 Proton

```
       SAMPLE                     SPECIAL
date    Jun 16 2015    temp              24.0
solvent       cdcl3    gain          not used
file /home/pharmd/~    spin                20
vnmrsys/data/auto_~    hst              0.008
2015.06.09/0026.fi~    pw90            12.750
                  d    alfa             6.600
    ACQUISITION               FLAGS
sw           6399.0    il                   n
at            2.049    in                   n
np            26218    dp                   y
fb             4000    hs                  nn
bs               32          PROCESSING
ss                2    fn               65536
d1            1.000           DISPLAY
nt               16    sp               253.5
ct               16    wp               930.9
    TRANSMITTER        rfl              763.7
tn               H1    rfp                  0
sfrq        399.949    rp               -98.2
tof           399.9    lp               -58.1
tpwr             50             PLOT
pw            6.375    wc                 250
     DECOUPLER         sc                   0
dn              C13    vs                 111
dof               0    th                   4
dm              nnn    ai   cdc  ph
dmm               c
dpwr             40
dmf           17100
```

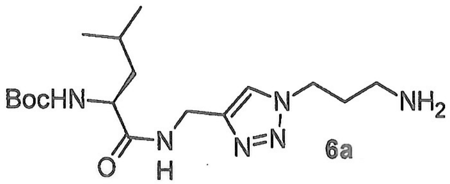

6a

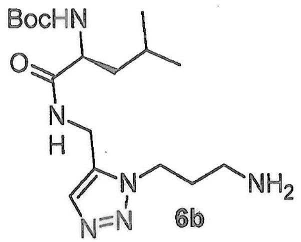

6b

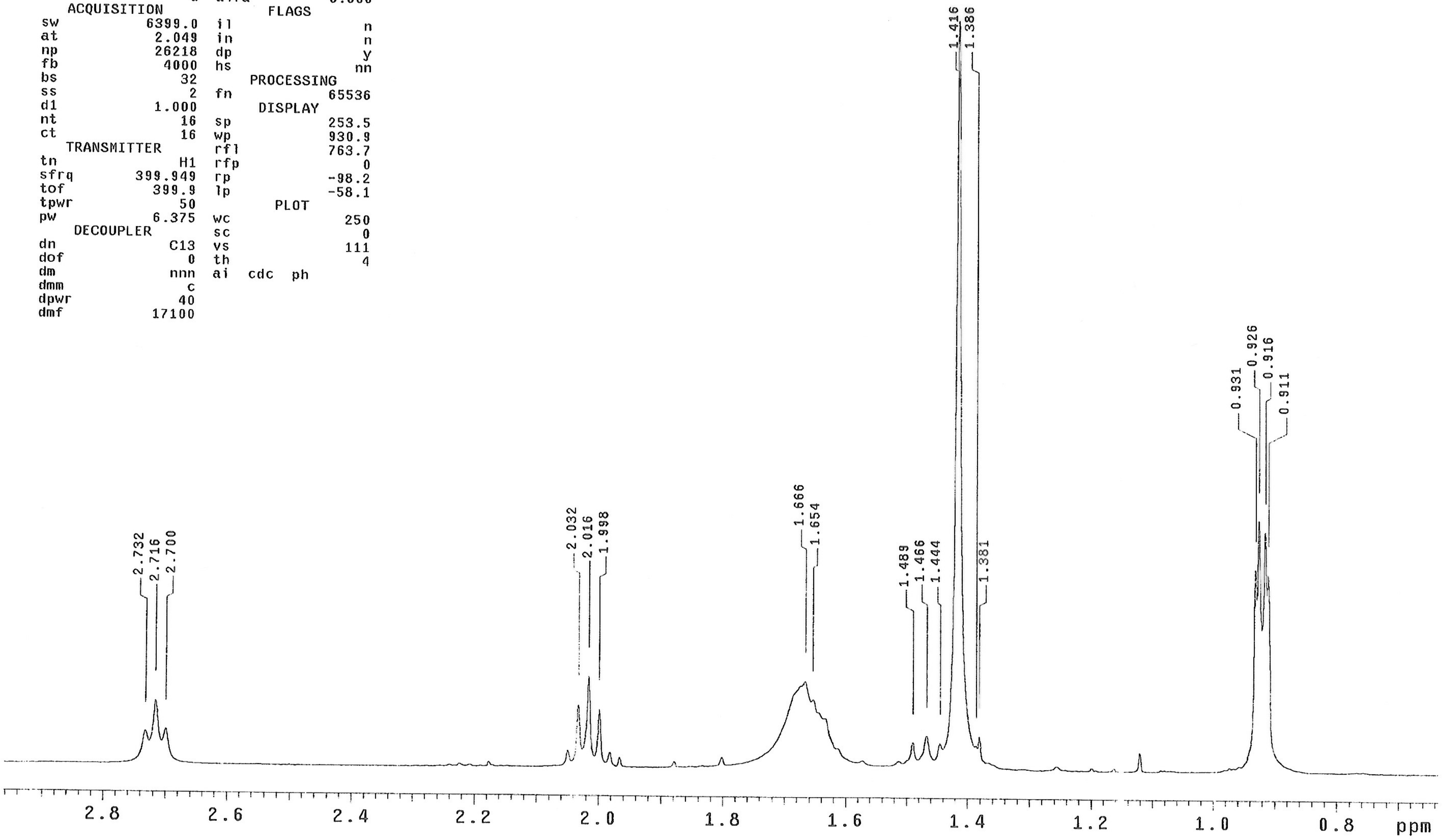

13-0203-011-21-09
[illegible] 06/26/15

13-0203-011-21-2
col 1 fr 17-21

exp1 Carbon

SAMPLE		SPECIAL	
date	Jun 25 2015	temp	24.0
solvent	cdcl3	gain	30
file	/home/pharmd/~	spin	20
vnmrsys/data/auto_~		hst	0.008
2015.06.09/0048.fi~		pw90	12.300
	d	alfa	6.600
ACQUISITION		FLAGS	
sw	24132.7	il	n
at	1.300	in	n
np	62768	dp	y
fb	13000	hs	nn
bs	64	PROCESSING	
d1	1.000	lb	0.50
nt	5000	fn	not used
ct	5000	DISPLAY	
TRANSMITTER		sp	-95.0
tn	C13	wp	17649.6
sfrq	100.577	rfl	1503.1
tof	1028.5	rfp	0
tpwr	51	rp	-52.6
pw	6.150	lp	-120.2
DECOUPLER		PLOT	
dn	H1	wc	250
dof	0	sc	0
dm	yyy	vs	83171
dmm	w	th	4
dpwr	33	ai	cdc ph
dmf	9300		

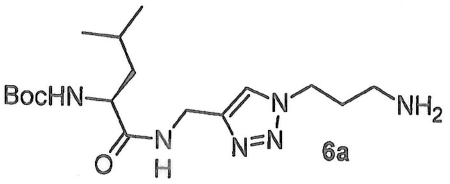

6a

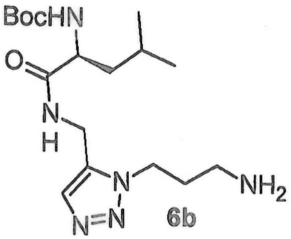

6b

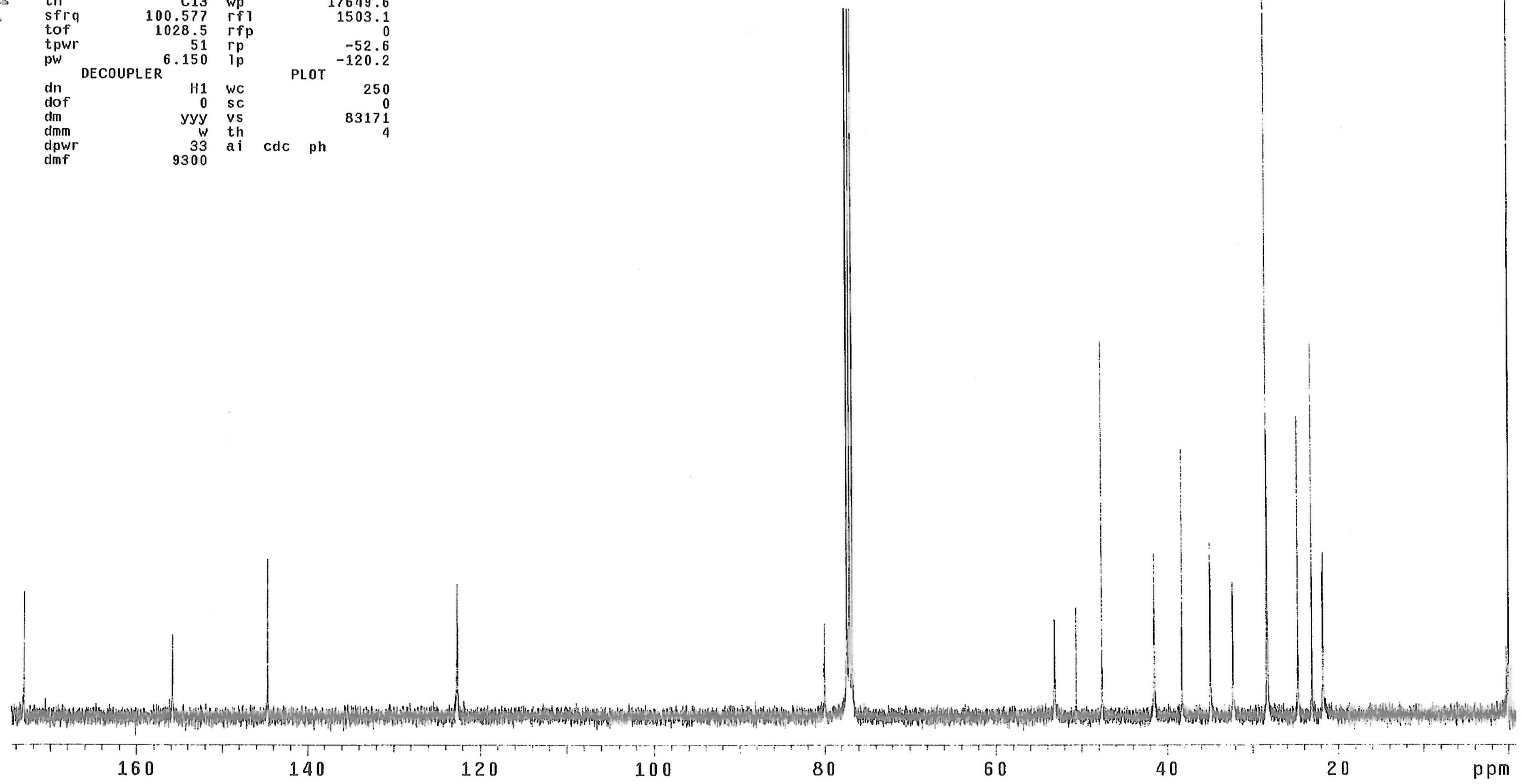

13-0203-011-21-2
col 1 fr 17-21

exp1 Carbon

SAMPLE		SPECIAL	
date	Jun 25 2015	temp	24.0
solvent	cdcl3	gain	30
file	/home/pharmd/~	spin	20
vnmrsys/data/auto_~		hst	0.008
2015.06.09/0048.fi~		pw90	12.300
	d	alfa	6.600
ACQUISITION		FLAGS	
sw	24132.7	il	n
at	1.300	in	n
np	62768	dp	y
fb	13000	hs	nn
bs	64	PROCESSING	
d1	1.000	lb	0.50
nt	5000	fn	not used
ct	5000	DISPLAY	
TRANSMITTER		sp	7923.7
tn	C13	wp	9630.1
sfrq	100.577	rfl	1503.1
tof	1028.5	rfp	0
tpwr	51	rp	-52.6
pw	6.150	lp	-120.2
DECOUPLER		PLOT	
dn	H1	wc	250
dof	0	sc	0
dm	yyy	vs	83171
dmm	w	th	9
dpwr	33	ai cdc ph	
dmf	9300		

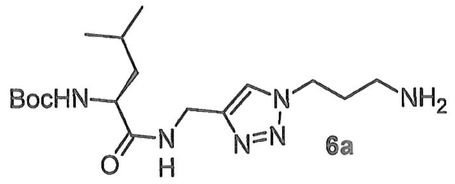

6a

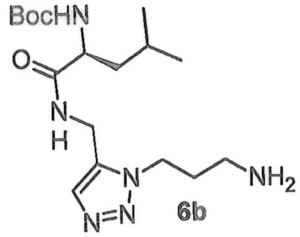

6b

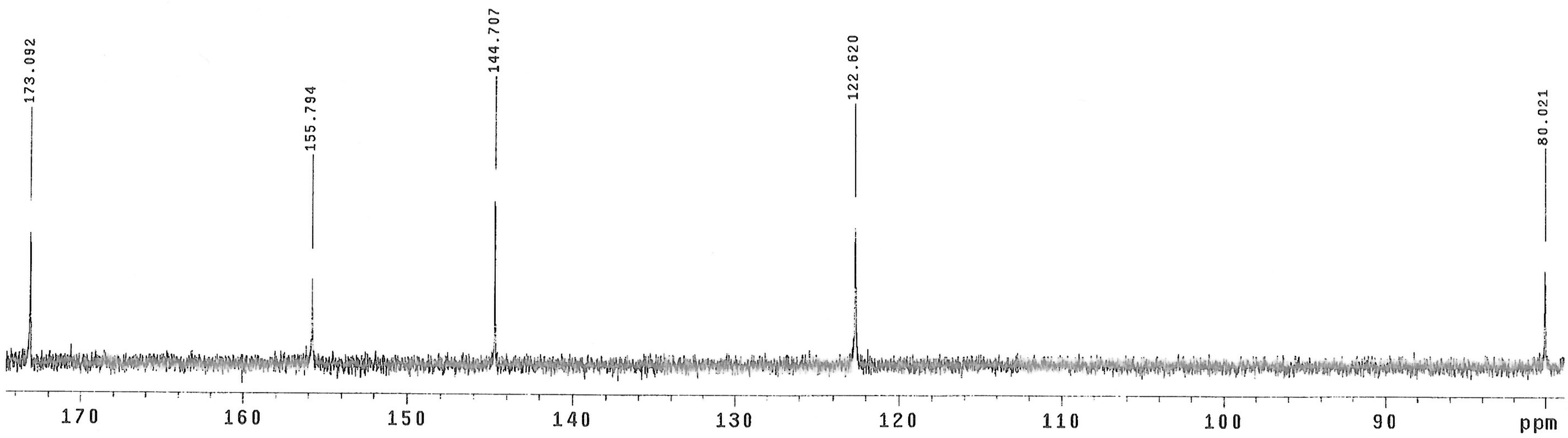

```
13-0203-011-21-2
col 1 fr 17-21

exp1  Carbon

      SAMPLE                 SPECIAL
date   Jun 25 2015   temp          24.0
solvent       cdcl3  gain            30
file /home/pharmd/~  spin            20
vnmrsys/data/auto_~  hst          0.008
2015.06.09/0048.fi~  pw90        12.300
                  d  alfa         6.600
   ACQUISITION              FLAGS
sw          24132.7  il               n
at            1.300  in               n
np            62768  dp               y
fb            13000  hs              nn
bs               64        PROCESSING
d1            1.000  lb            0.50
nt             5000  fn        not used
ct             5000         DISPLAY
   TRANSMITTER       sp          1950.2
tn              C13  wp          3562.3
sfrq        100.577  rfl         1503.1
tof          1028.5  rfp              0
tpwr             51  rp           -52.6
pw            6.150  lp          -120.2
    DECOUPLER                PLOT
dn               H1  wc             250
dof               0  sc               0
dm              yyy  vs           57990
dmm               w  th               9
dpwr             33  ai   cdc  ph
dmf            9300
```

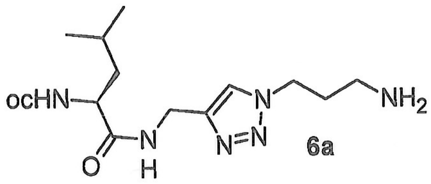

6a

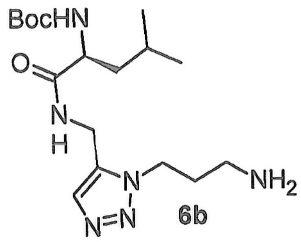

6b

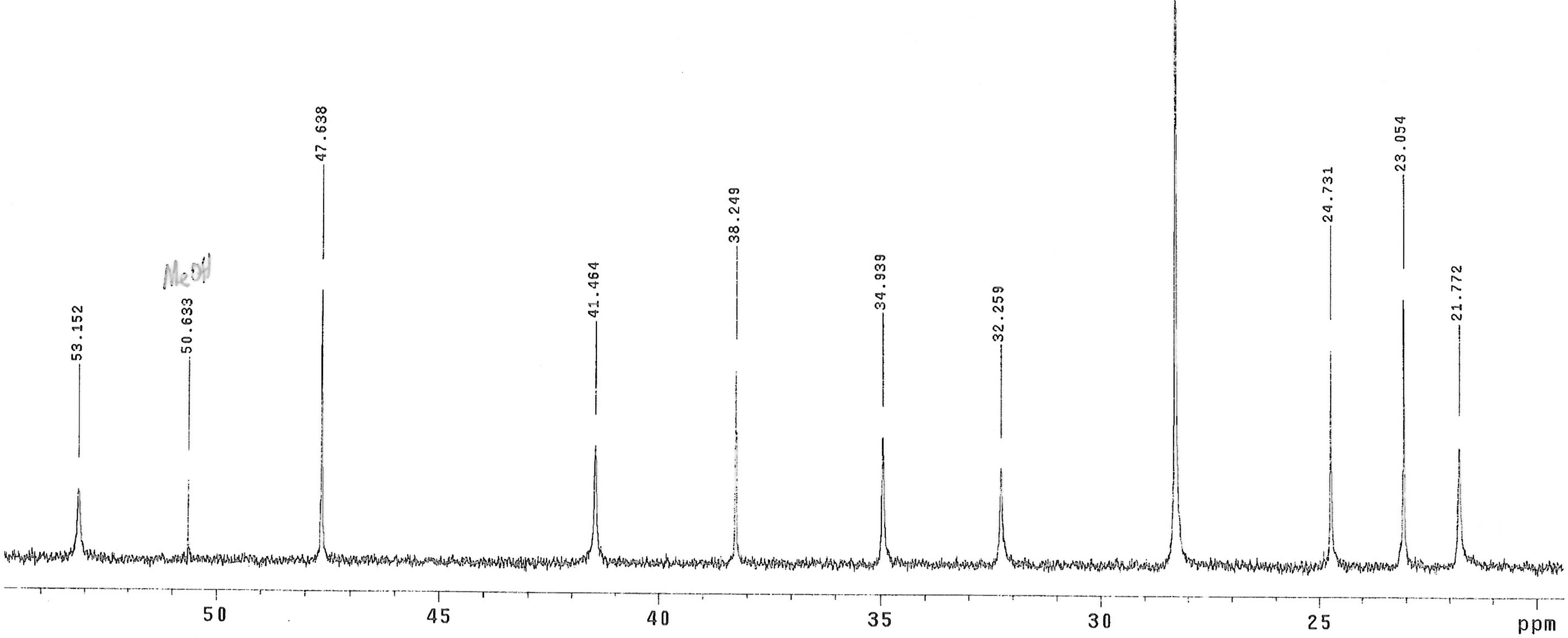

Print of window 80: MS Spectrum
13-0203-011-21-1
col 1, fr 17-21

==

Injection Date	: 6/24/2015 12:07:45 PM	Seq. Line :	1
Sample Name	: 130203011-21-1	Location :	Vial 17
.. Operator	: Synthesis	Inj :	1
Acq. Instrument	: Instrument 1	Inj Volume :	5 μl
Sequence File	: C:\HPCHEM\1\SEQUENCE\SYN2013.S		
Method	: C:\HPCHEM\1\METHODS\GENPOS2.M		
Last changed	: 11/8/2014 12:44:50 PM by Synthesis Group		

General use pos

MS Spectrum

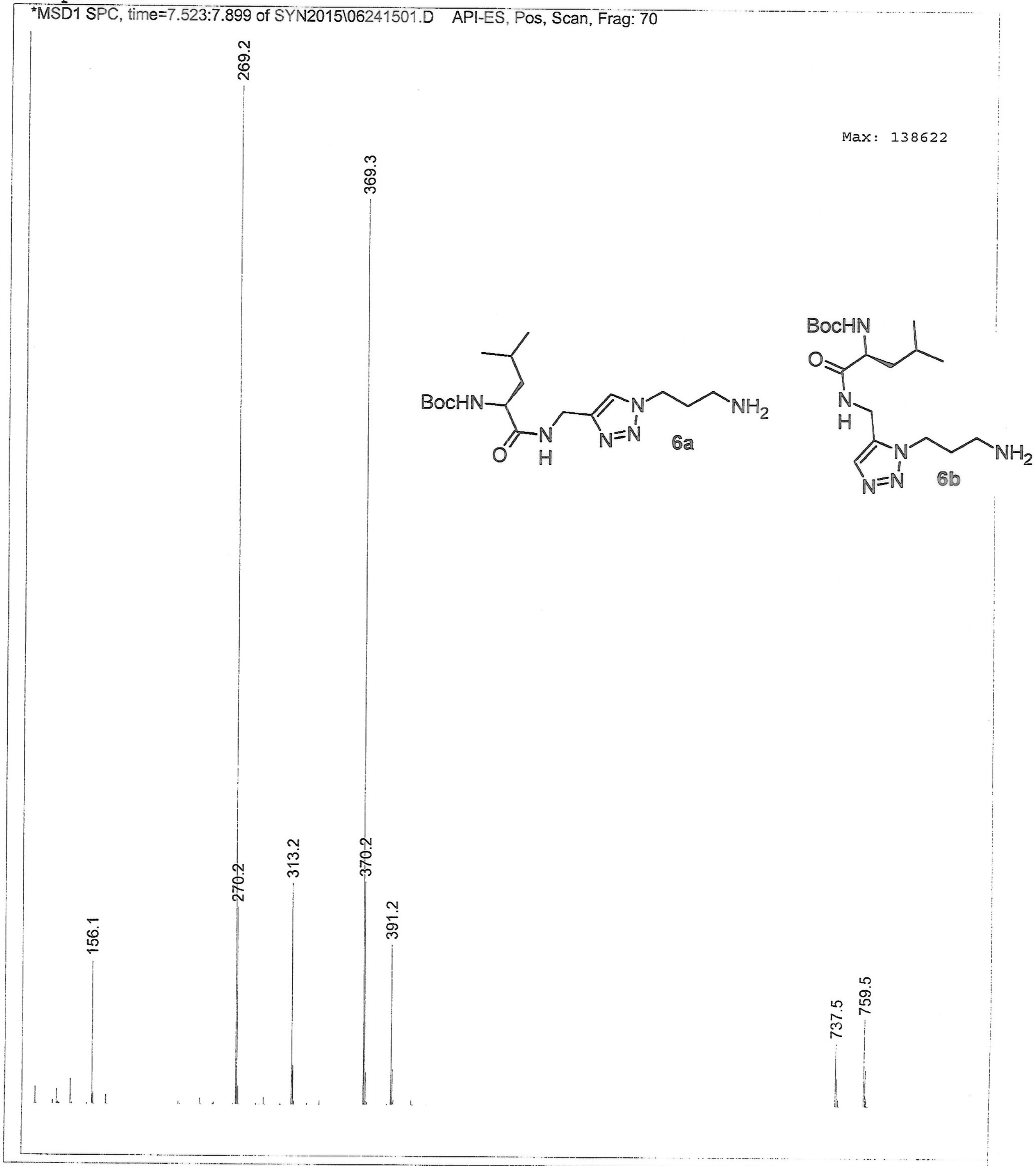

13-0203-011-24-32
W. [illegible] 07/01/15

```
13-0203-011-24-2
col 2 fr 13

exp1  Proton

      SAMPLE                    SPECIAL
date    Jul  1 2015   temp            24.0
solvent       cdcl3   gain        not used
file /home/pharmd/~   spin              20
vnmrsys/data/auto_~   hst            0.008
2015.06.09/0081.fi~   pw90          11.500
                  d   alfa           6.600
   ACQUISITION               FLAGS
sw           6399.0   il                 n
at            2.049   in                 n
np            26218   dp                 y
fb             4000   hs                nn
bs               32         PROCESSING
ss                2   fn             65536
d1            1.000          DISPLAY
nt               16   sp             275.3
ct               16   wp            2808.9
   TRANSMITTER        rfl            775.7
tn               H1   rfp                0
sfrq        399.949   rp             -93.0
tof           399.9   lp             -50.4
tpwr             51           PLOT
pw            5.750   wc               250
    DECOUPLER         sc                 0
dn              C13   vs               106
dof               0   th                 3
dm              nnn   ai   cdc  ph
dmm               c
dpwr             40
dmf           17100
```

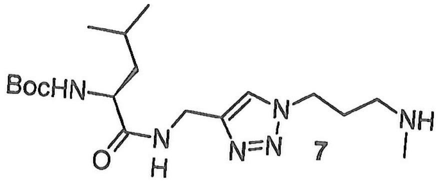

7

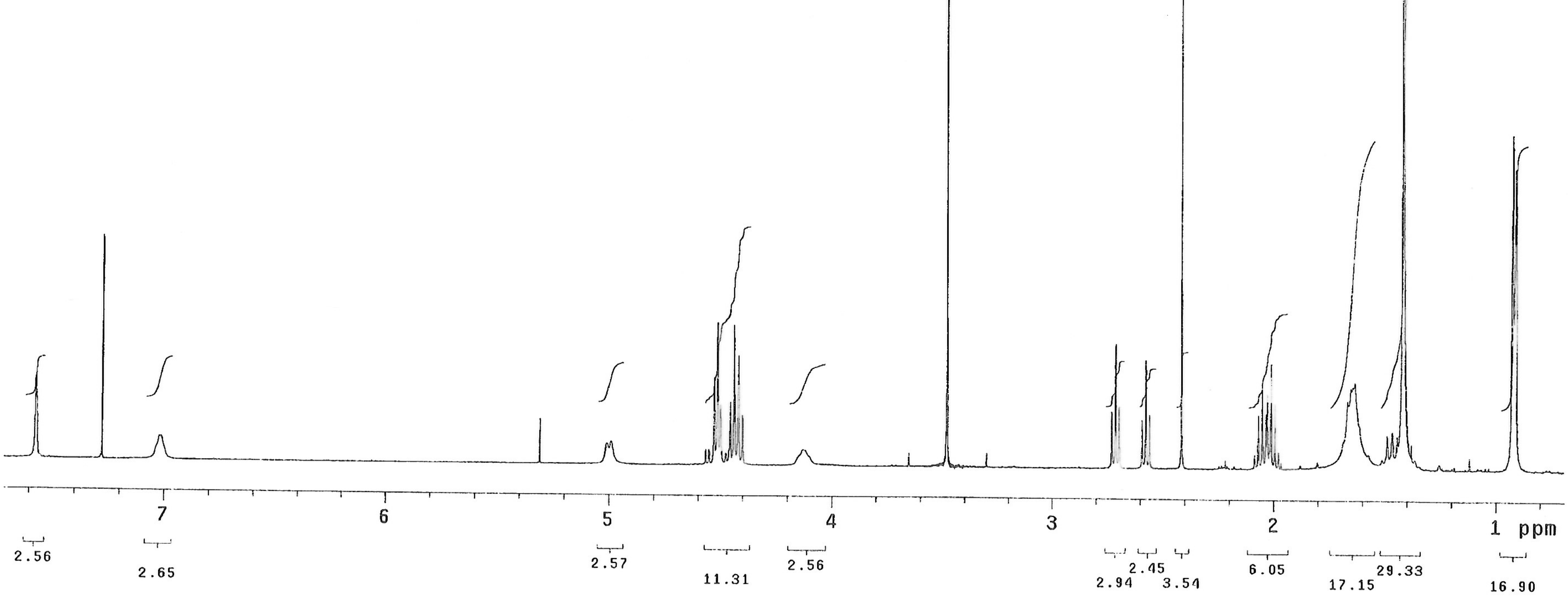

```
13-0203-011-24-2
col 2 fr 13

exp1  Proton

       SAMPLE                  SPECIAL
date    Jul  1 2015   temp            24.0
solvent       cdcl3   gain        not used
file /home/pharmd/~   spin              20
vnmrsys/data/auto_~   hst            0.008
2015.06.09/0081.fi~   pw90          11.500
                  d   alfa           6.600
    ACQUISITION                FLAGS
sw           6399.0   il                 n
at            2.049   in                 n
np            26218   dp                 y
fb             4000   hs                nn
bs               32          PROCESSING
ss                2   fn             65536
d1            1.000            DISPLAY
nt               16   sp            2681.6
ct               16   wp             530.2
    TRANSMITTER       rfl            775.7
tn               H1   rfp                0
sfrq        399.949   rp             -93.0
tof           399.9   lp             -50.4
tpwr             51             PLOT
pw            5.750   wc               250
    DECOUPLER         sc                 0
dn              C13   vs               369
dof               0   th                10
dm              nnn   ai   cdc  ph
dmm               c
dpwr             40
dmf           17100
```

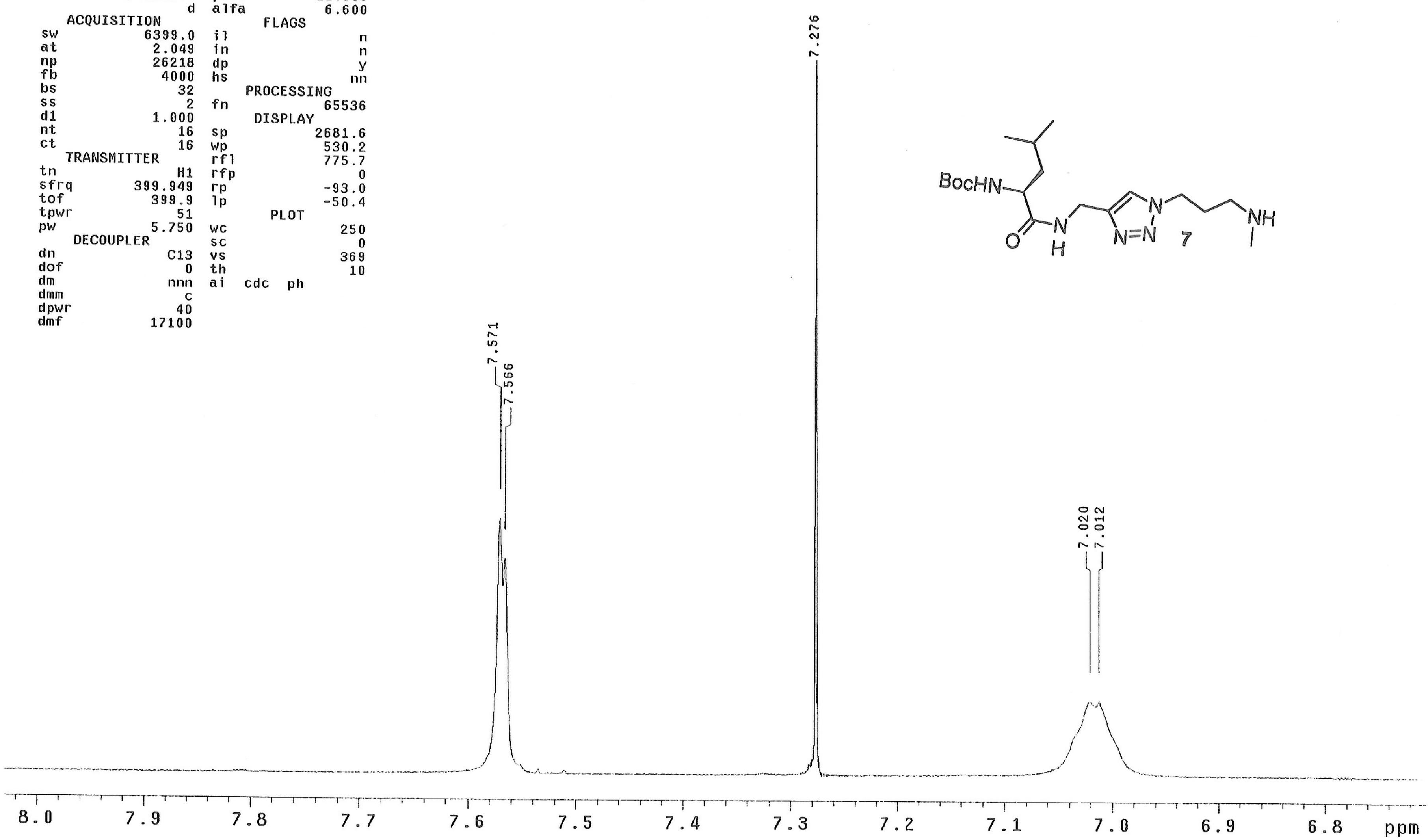

7

13-0203-011-24-2
col 2 fr 13

exp1 Proton

SAMPLE		SPECIAL	
date	Jul 1 2015	temp	24.0
solvent	cdcl3	gain	not used
file	/home/pharmd/~	spin	20
vnmrsys/data/auto_~		hst	0.008
2015.06.09/0081.fi~		pw90	11.500
	d	alfa	6.600
ACQUISITION		FLAGS	
sw	6399.0	il	n
at	2.049	in	n
np	26218	dp	y
fb	4000	hs	nn
bs	32	PROCESSING	
ss	2	fn	65536
d1	1.000	DISPLAY	
nt	16	sp	1522.6
ct	16	wp	756.1
TRANSMITTER		rfl	775.7
tn	H1	rfp	0
sfrq	399.949	rp	-93.0
tof	399.9	lp	-50.4
tpwr	51	PLOT	
pw	5.750	wc	250
DECOUPLER		sc	0
dn	C13	vs	490
dof	0	th	6
dm	nnn	ai cdc ph	
dmm	c		
dpwr	40		
dmf	17100		

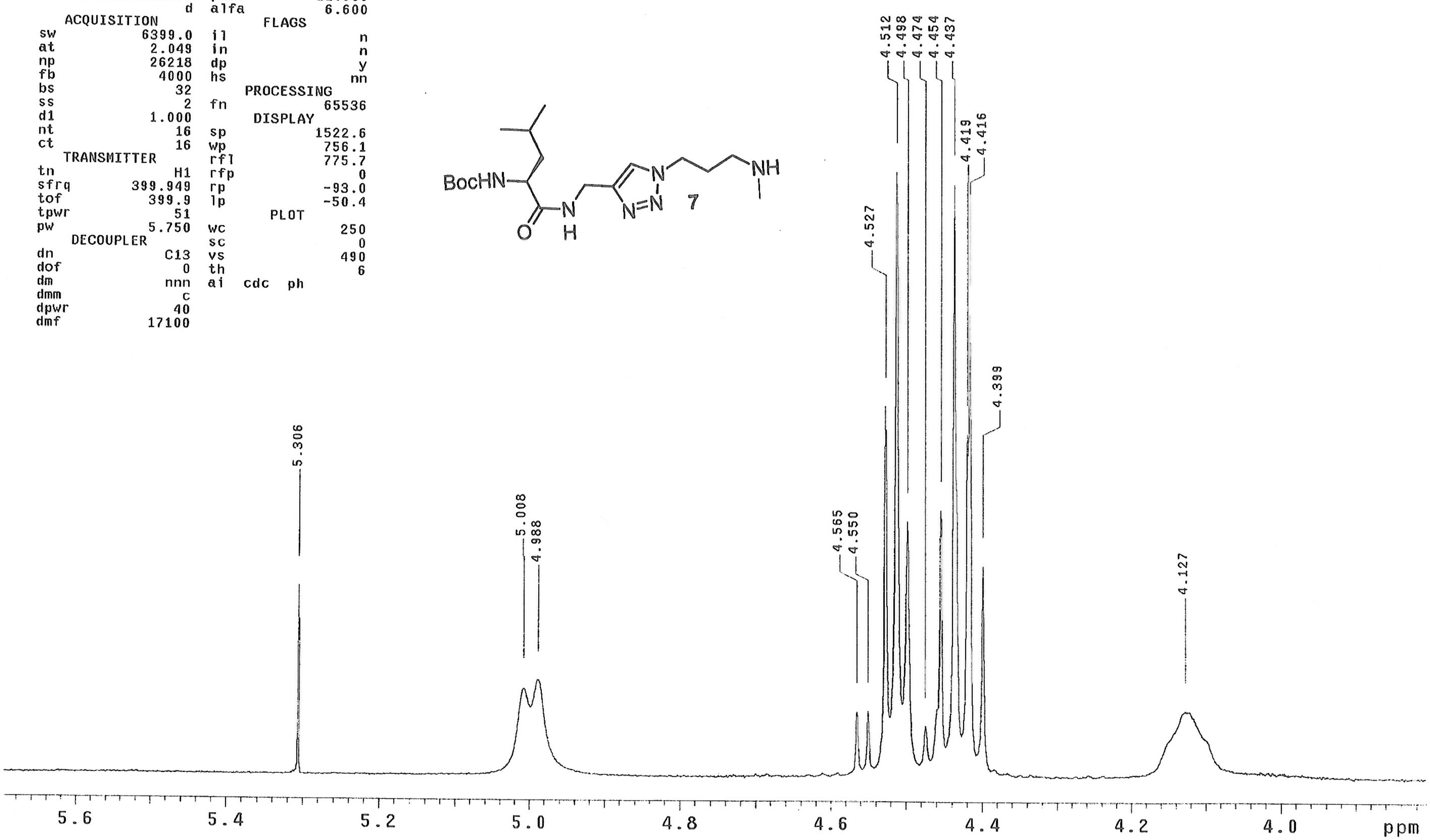

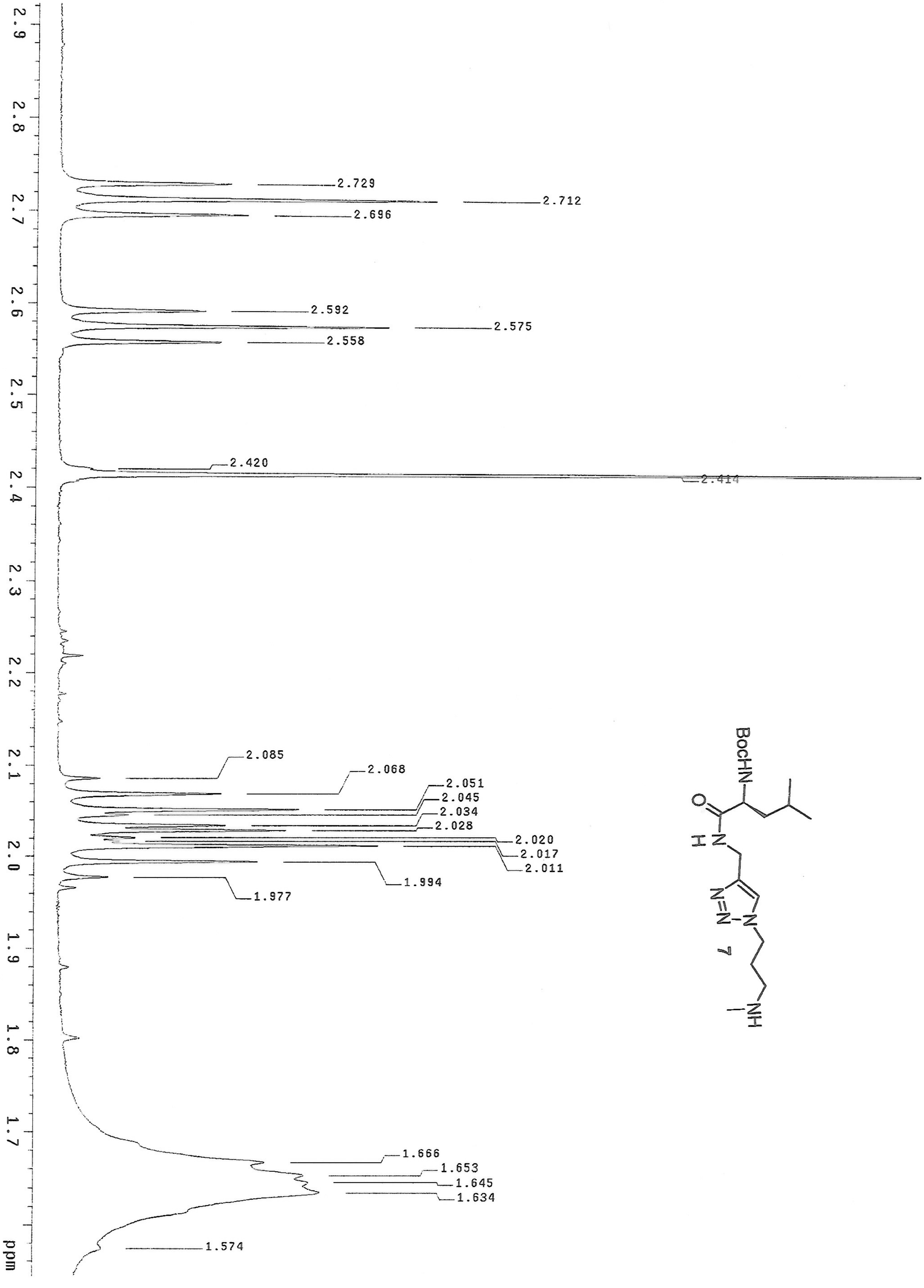
2.9
2.8
2.7
2.6
2.5
2.4
2.3
2.2
2.1
2.0
1.9
1.8
1.7
ppm
2.729
2.712
2.696
2.592
2.575
2.558
2.420
2.414
2.085
2.068
2.051
2.045
2.034
2.028
2.020
2.017
2.011
1.994
1.977
1.666
1.653
1.645
1.634
1.574
BocHN
O
N
H
N=N
N
NH
7

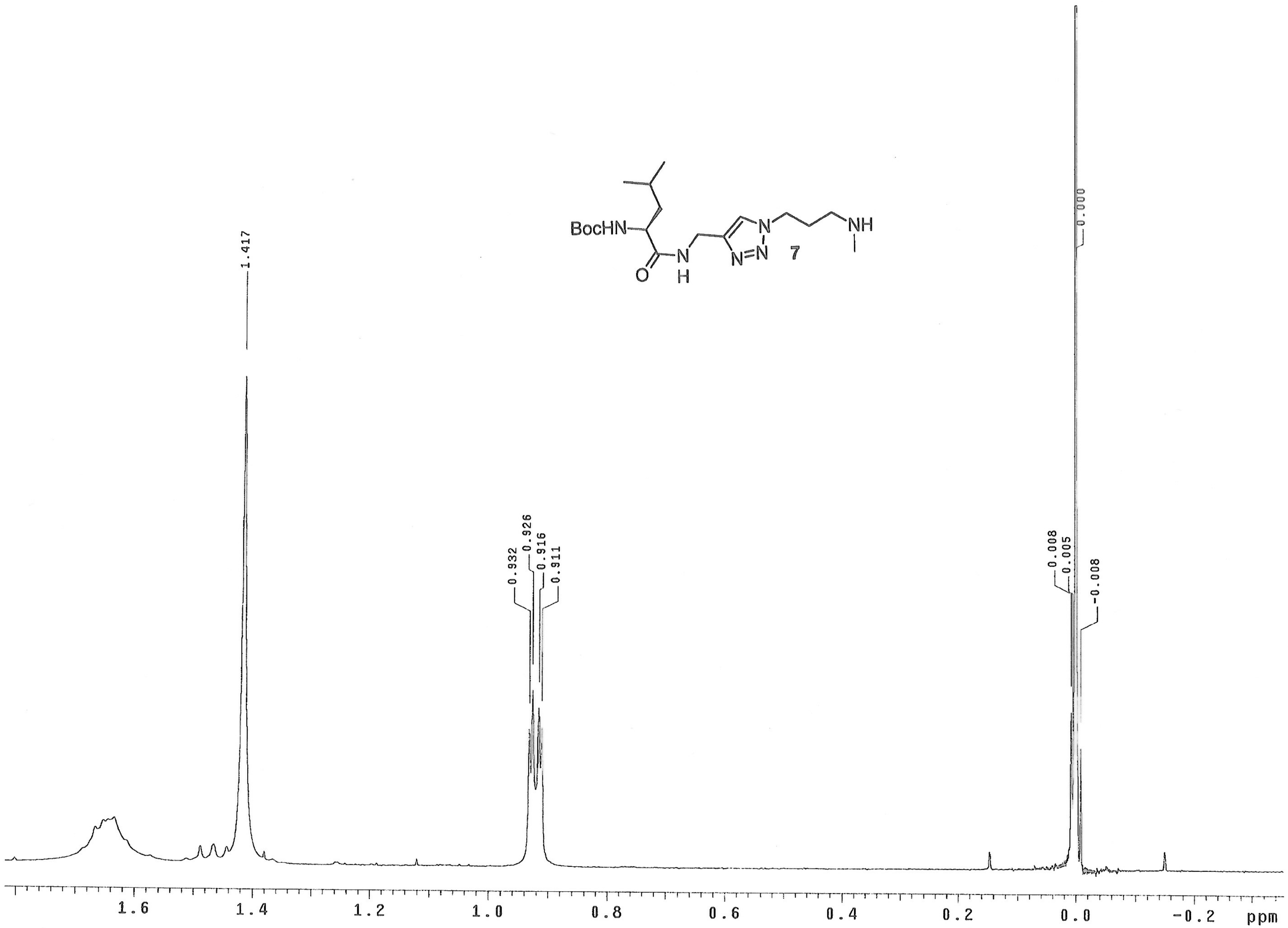
BocHN
O
N
H
N=N
N
NH
7
1.417
0.932
0.926
0.916
0.911
0.008
0.005
0.000
-0.008
1.6
1.4
1.2
1.0
0.8
0.6
0.4
0.2
0.0
-0.2
ppm

13-0203-011-24-37
W. Carter 07/08/15

13-0203-011-24-2

expl Carbon

SAMPLE		SPECIAL	
date	Jul 8 2015	temp	24.0
solvent	cdcl3	gain	30
file	/home/pharmd/~	spin	20
vnmrsys/data/auto_~		hst	0.008
2015.06.09/0099.fi~		pw90	12.300
	d	alfa	6.600
ACQUISITION		FLAGS	
sw	24132.7	il	n
at	1.300	in	n
np	62768	dp	y
fb	13000	hs	nn
bs	64	PROCESSING	
d1	1.000	lb	0.50
nt	1000	fn	not used
ct	1000	DISPLAY	
TRANSMITTER		sp	1979.2
tn	C13	wp	15602.9
sfrq	100.577	rfl	9252.9
tof	1028.5	rfp	7759.7
tpwr	51	rp	-52.9
pw	6.150	lp	-133.8
DECOUPLER		PLOT	
dn	H1	wc	250
dof	0	sc	0
dm	yyy	vs	28440
dmm	w	th	3
dpwr	33	ai	cdc ph
dmf	9300		

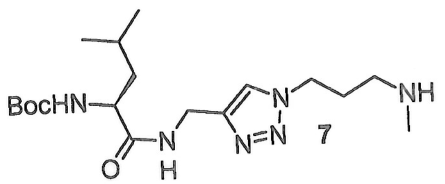

7

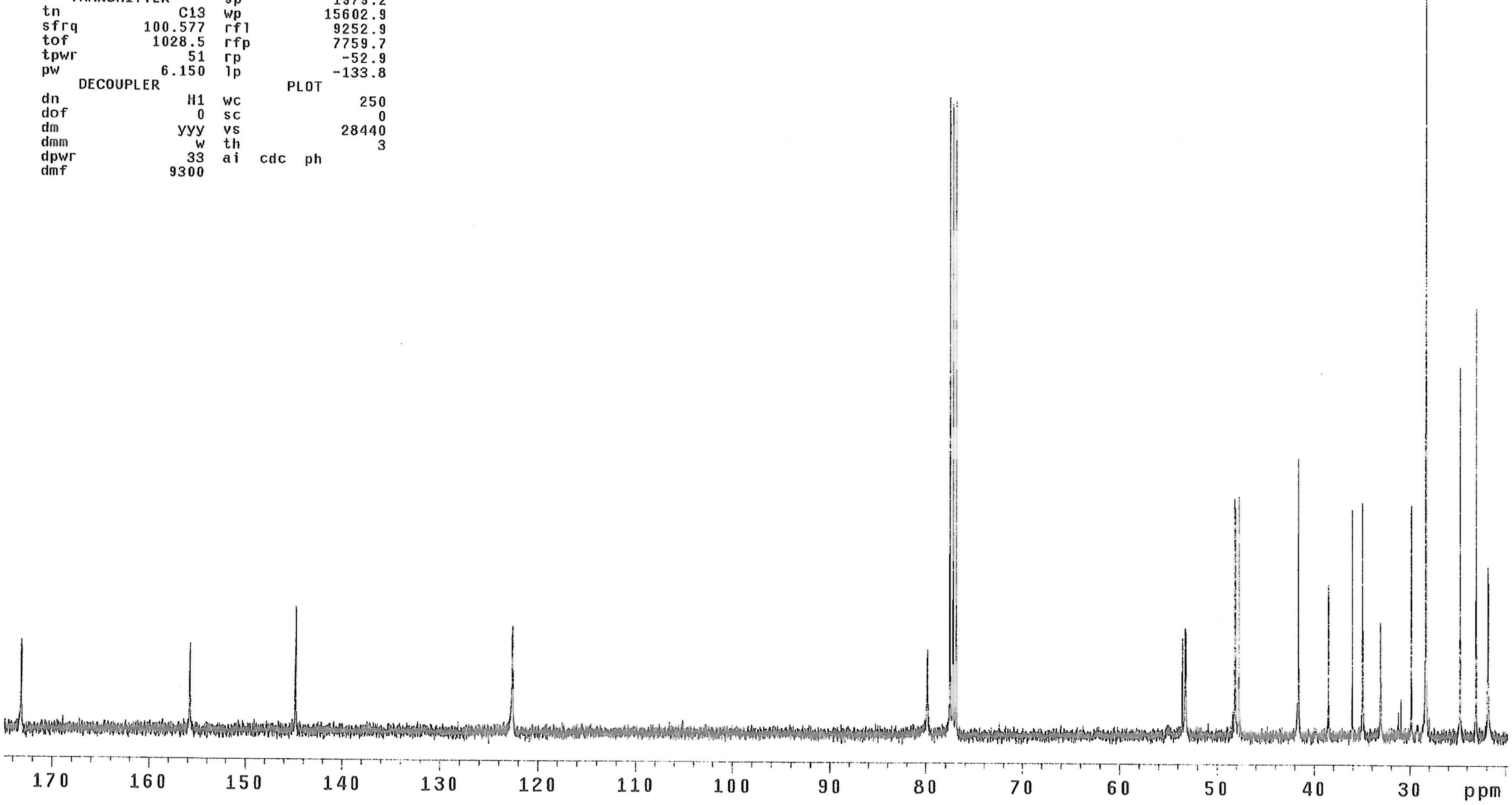

13-0203-011-24-2

exp1 Carbon

```
      SAMPLE                  SPECIAL
date    Jul  8 2015   temp          24.0
solvent       cdcl3   gain            30
file /home/pharmd/~   spin            20
vnmrsys/data/auto_~   hst          0.008
2015.06.09/0099.fl~   pw90        12.300
                  d   alfa         6.600
   ACQUISITION                FLAGS
sw          24132.7   il               n
at            1.300   in               n
np            62768   dp               y
fb            13000   hs              nn
bs               64         PROCESSING
d1            1.000   lb            0.50
nt             1000   fn        not used
ct             1000          DISPLAY
   TRANSMITTER        sp          7619.1
tn              C13   wp          9962.3
sfrq        100.577   rfl         9252.9
tof          1028.5   rfp         7759.7
tpwr             51   rp           -52.9
pw            6.150   lp          -133.8
    DECOUPLER                 PLOT
dn               H1   wc             250
dof               0   sc               0
dm              yyy   vs           28440
dmm               w   th              10
dpwr             33   ai   cdc  ph
dmf            9300
```

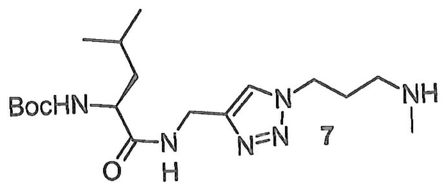

7

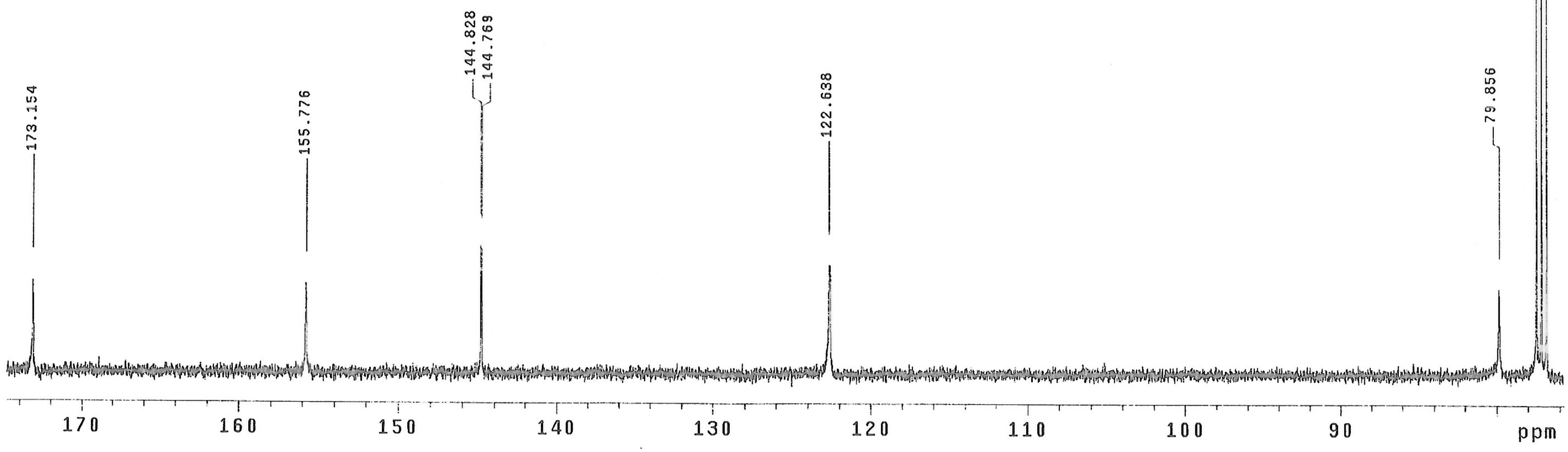

13-0203-011-24-2

exp1 Carbon

SAMPLE		SPECIAL	
date	Jul 8 2015	temp	24.0
solvent	cdcl3	gain	30
file /home/pharmd/~		spin	20
vnmrsys/data/auto_~		hst	0.008
2015.06.09/0099.fi~		pw90	12.300
	d	alfa	6.600
ACQUISITION		FLAGS	
sw	24132.7	il	n
at	1.300	in	n
np	62768	dp	y
fb	13000	hs	nn
bs	64	PROCESSING	
d1	1.000	lb	0.50
nt	1000	fn	not used
ct	1000	DISPLAY	
TRANSMITTER		sp	2027.1
tn	C13	wp	3520.3
sfrq	100.577	rfl	9252.9
tof	1028.5	rfp	7759.7
tpwr	51	rp	-52.9
pw	6.150	lp	-133.8
DECOUPLER		PLOT	
dn	H1	wc	250
dof	0	sc	0
dm	yyy	vs	28440
dmm	w	th	10
dpwr	33	ai	cdc ph
dmf	9300		

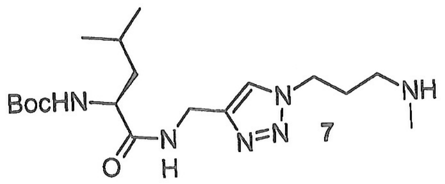

7

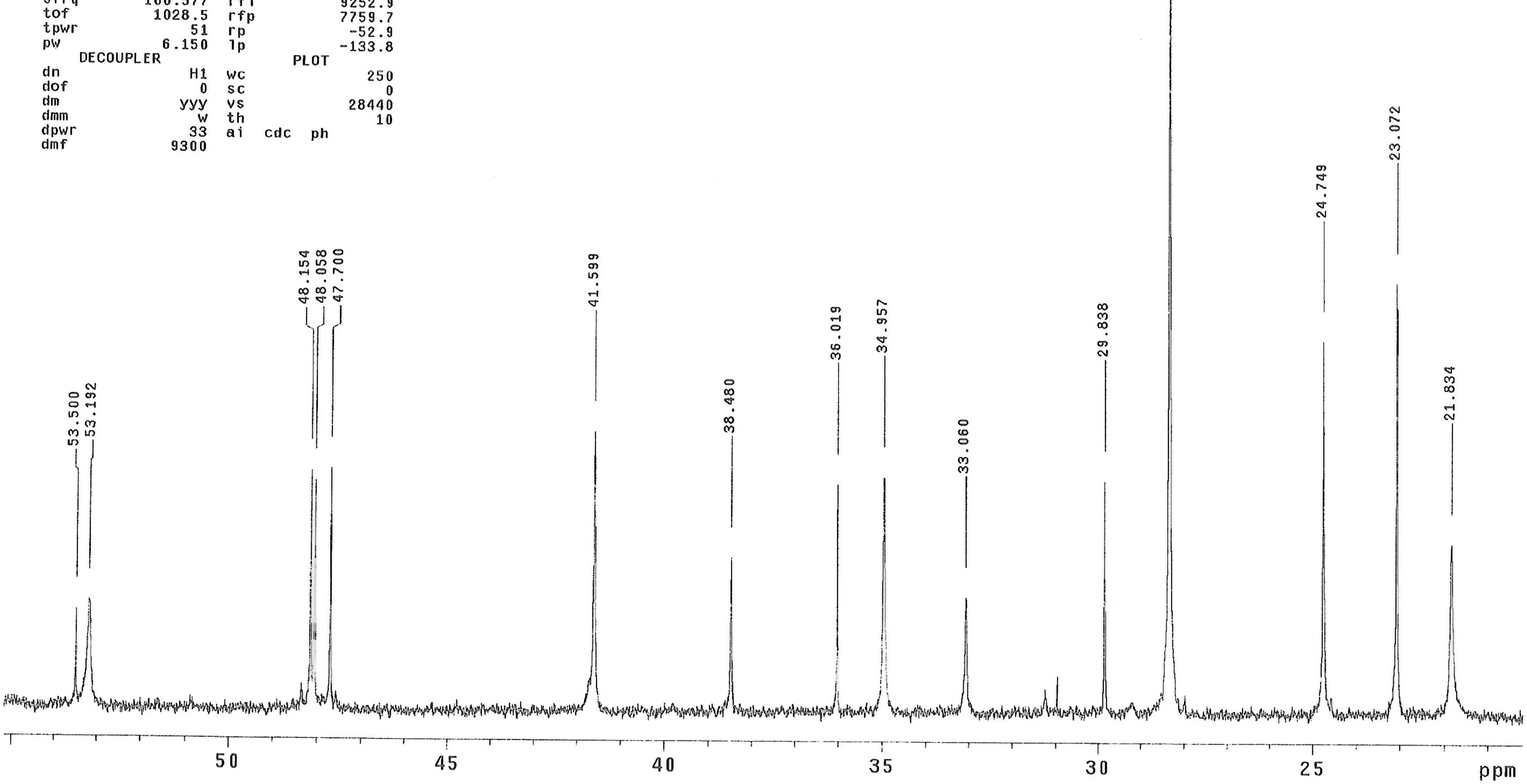

Print of window 80: MS Spectrum
13-0203-011-24-4
col 2 fr 14

==

Injection Date	: 7/1/2015 12:25:37 PM	Seq. Line :	2
Sample Name	: 130203011-24-4	Location :	Vial 12
Acq. Operator	: Synthesis	Inj :	1
Acq. Instrument	: Instrument 1	Inj Volume :	2 µl
Method	: C:\HPCHEM\1\METHODS\GENPOS-S.M		
Last changed	: 3/23/2015 2:38:34 PM by Ming		

General use pos short run time

MS Spectrum

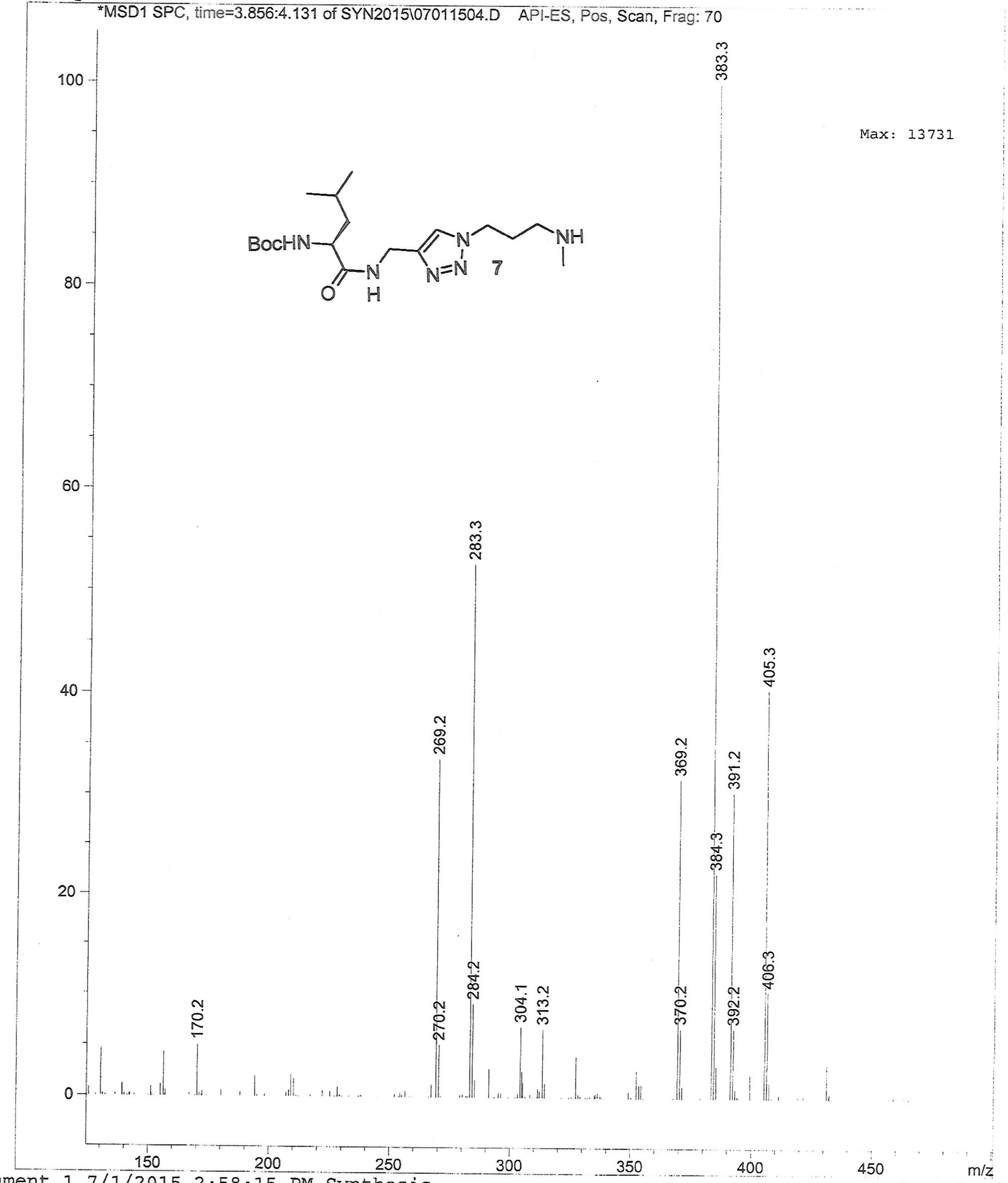

13-0203-011-25-36
W. Gantz 07/07/15

13-0203-011-25-1
col 1, fr 7 & 8

exp1 Proton

SAMPLE		SPECIAL	
date	Jul 7 2015	temp	24.0
solvent	cdcl3	gain	not used
file	/home/pharmd/~	spin	20
vnmrsys/data/auto_~		hst	0.008
2015.06.09/0093.fi~		pw90	11.500
	d	alfa	6.600
ACQUISITION		FLAGS	
sw	6399.0	il	n
at	2.049	in	n
np	26218	dp	y
fb	4000	hs	nn
bs	32	PROCESSING	
ss	2	fn	65536
d1	1.000	DISPLAY	
nt	16	sp	248.6
ct	16	wp	3668.4
TRANSMITTER		rfl	753.4
tn	H1	rfp	0
sfrq	399.949	rp	-82.4
tof	399.9	lp	-49.4
tpwr	51	PLOT	
pw	5.750	wc	250
DECOUPLER		sc	0
dn	C13	vs	266
dof	0	th	3
dm	nnn	ai cdc ph	
dmm	c		
dpwr	40		
dmf	17100		

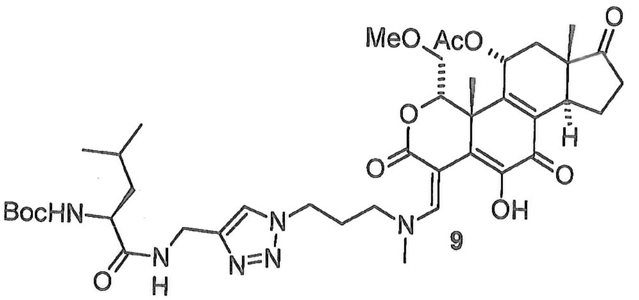

9

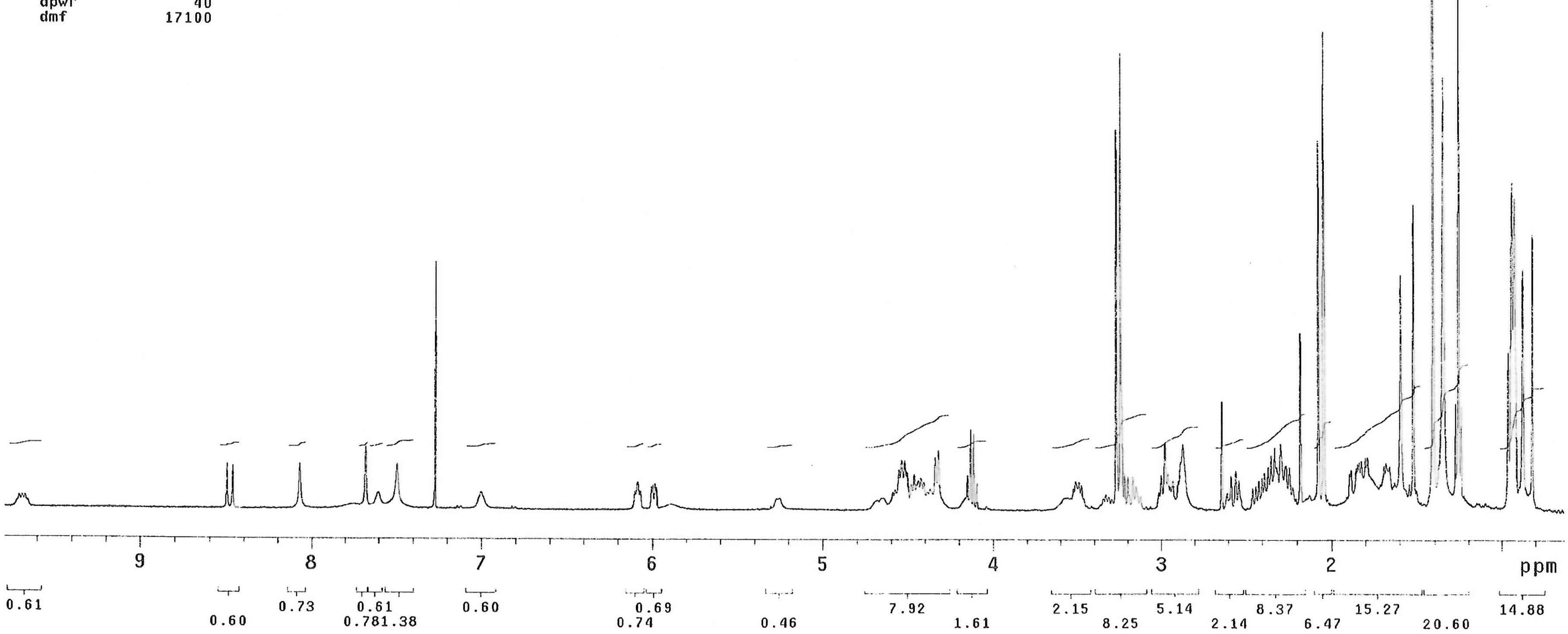

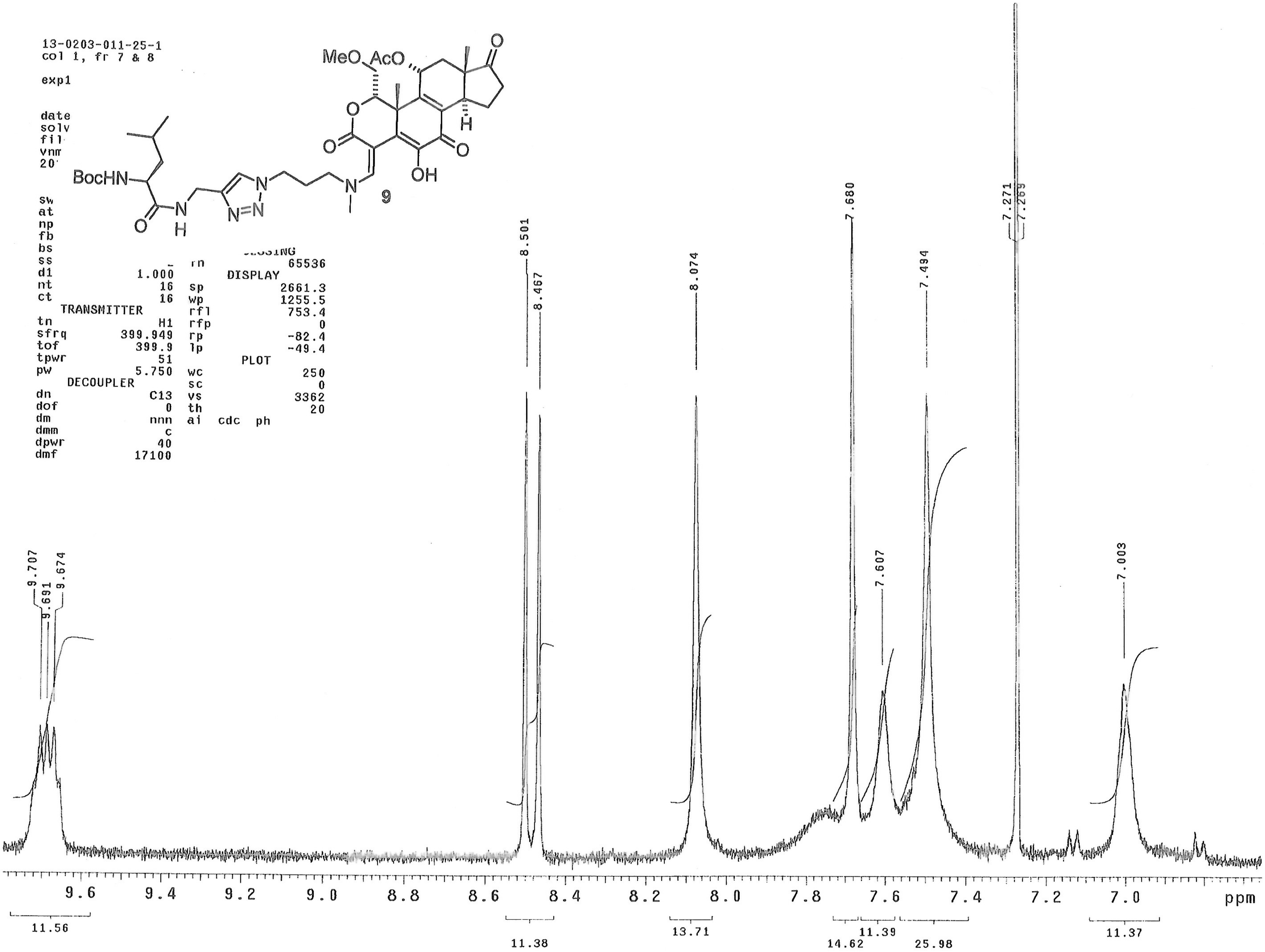

13-0203-011-25-1
col 1, fr 7 & 8
exp1
date
solv
fil
vnmr
20
sw
at
np
fb
bs
ss
d1 1.000
nt 16
ct 16
TRANSMITTER
tn H1
sfrq 399.949
tof 399.9
tpwr 51
pw 5.750
DECOUPLER
dn C13
dof 0
dm nnn
dmm c
dpwr 40
dmf 17100
rn 65536
DISPLAY
sp 2661.3
wp 1255.5
rfl 753.4
rfp 0
rp -82.4
lp -49.4
PLOT
wc 250
sc 0
vs 3362
th 20
ai cdc ph
BocHN
MeO
AcO
OH
9
9.707
9.691
9.674
8.501
8.467
8.074
7.680
7.607
7.494
7.271
7.269
7.003
9.6
9.4
9.2
9.0
8.8
8.6
8.4
8.2
8.0
7.8
7.6
7.4
7.2
7.0
ppm
11.56
11.38
13.71
14.62
11.39
25.98
11.37

13-0203-011-25-1
col 1, fr 7 & 8

exp1 Proton

SAMPLE		SPECIAL	
date	Jul 7 2015	temp	24.0
solvent	cdcl3	gain	not used
file /home/pharmd/~		spin	20
vnmrsys/data/auto_~		hst	0.008
2015.06.09/0093.fi~		pw90	11.500
	d	alfa	6.600
ACQUISITION		FLAGS	
sw	6399.0	il	n
at	2.049	in	n
np	26218	dp	y
fb	4000	hs	nn
bs	32	PROCESSING	
ss	2	fn	65536
d1	1.000	DISPLAY	
nt	16	sp	1985.6
ct	16	wp	616.5
TRANSMITTER		rfl	753.4
tn	H1	rfp	0
sfrq	399.949	rp	-82.4
tof	399.9	lp	-49.4
tpwr	51	PLOT	
pw	5.750	wc	250
DECOUPLER		sc	0
dn	C13	vs	3362
dof	0	th	19
dm	nnn	ai cdc ph	
dmm	c		
dpwr	40		
dmf	17100		

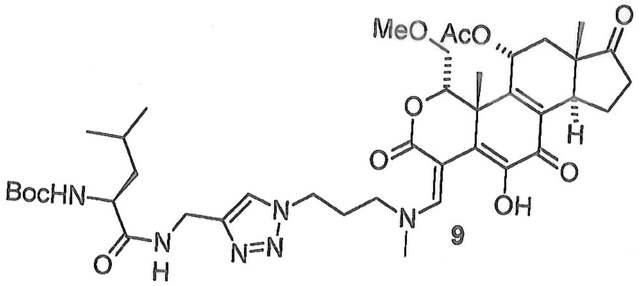

9

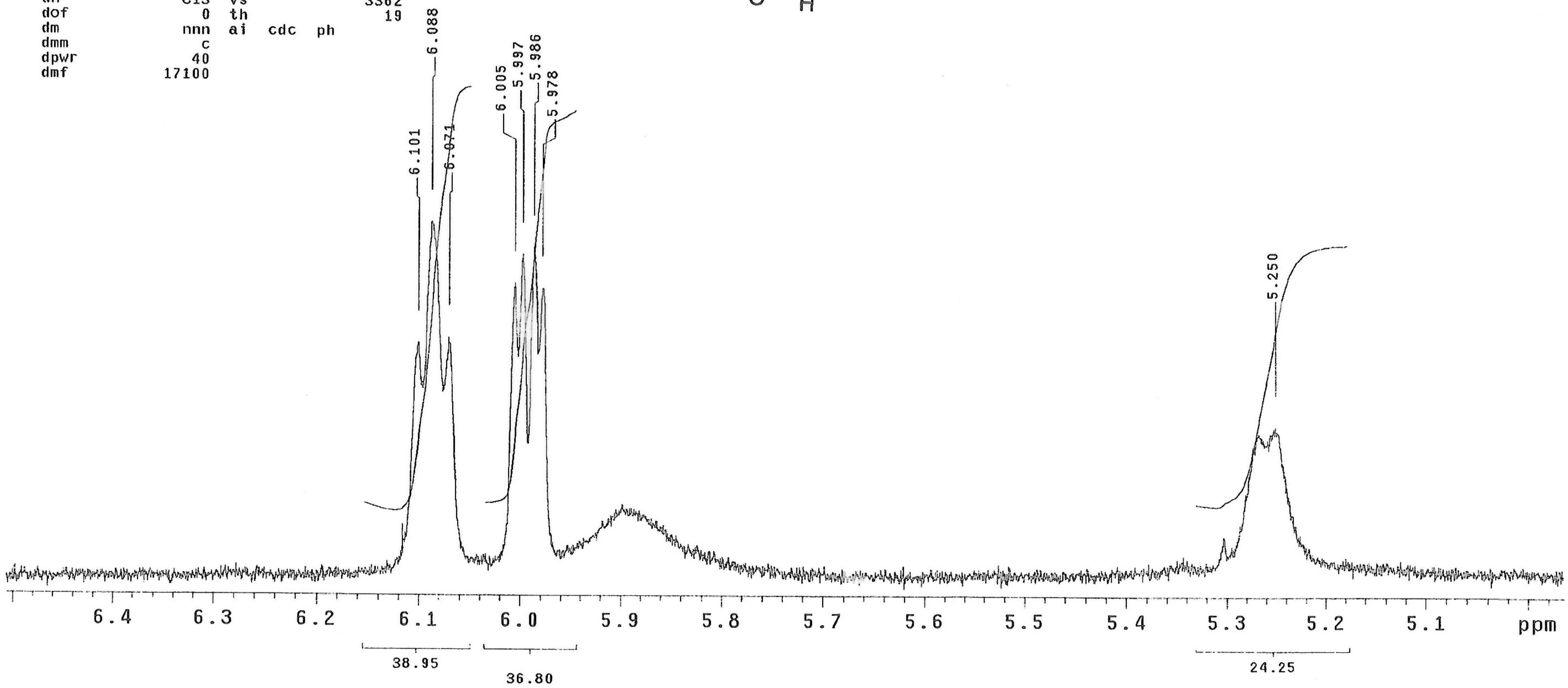

13-0203-011-25-1
col 1, fr 7 & 8

exp1 Proton

SAMPLE		SPECIAL	
date	Jul 7 2015	temp	24.0
solvent	cdcl3	gain	not used
file	/home/pharmd/~	spin	20
vnmrsys/data/auto_~		hst	0.008
2015.06.09/0093.fi~		pw90	11.500
	d	alfa	6.600
ACQUISITION		FLAGS	
sw	6399.0	il	n
at	2.049	in	n
np	26218	dp	y
fb	4000	hs	nn
bs	32	PROCESSING	
ss	2	fn	65536
d1	1.000	DISPLAY	
nt	16	sp	1557.6
ct	16	wp	433.5
TRANSMITTER		rfl	753.4
tn	H1	rfp	0
sfrq	399.949	rp	-82.4
tof	399.9	lp	-49.4
tpwr	51	PLOT	
pw	5.750	wc	250
DECOUPLER		sc	0
dn	C13	vs	1987
dof	0	th	19
dm	nnn	ai	cdc ph
dmm	c		
dpwr	40		
dmf	17100		

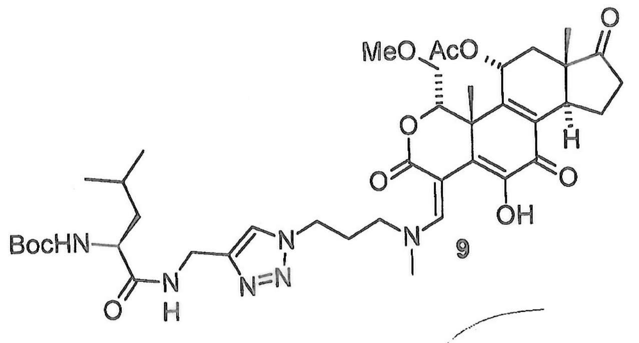

9

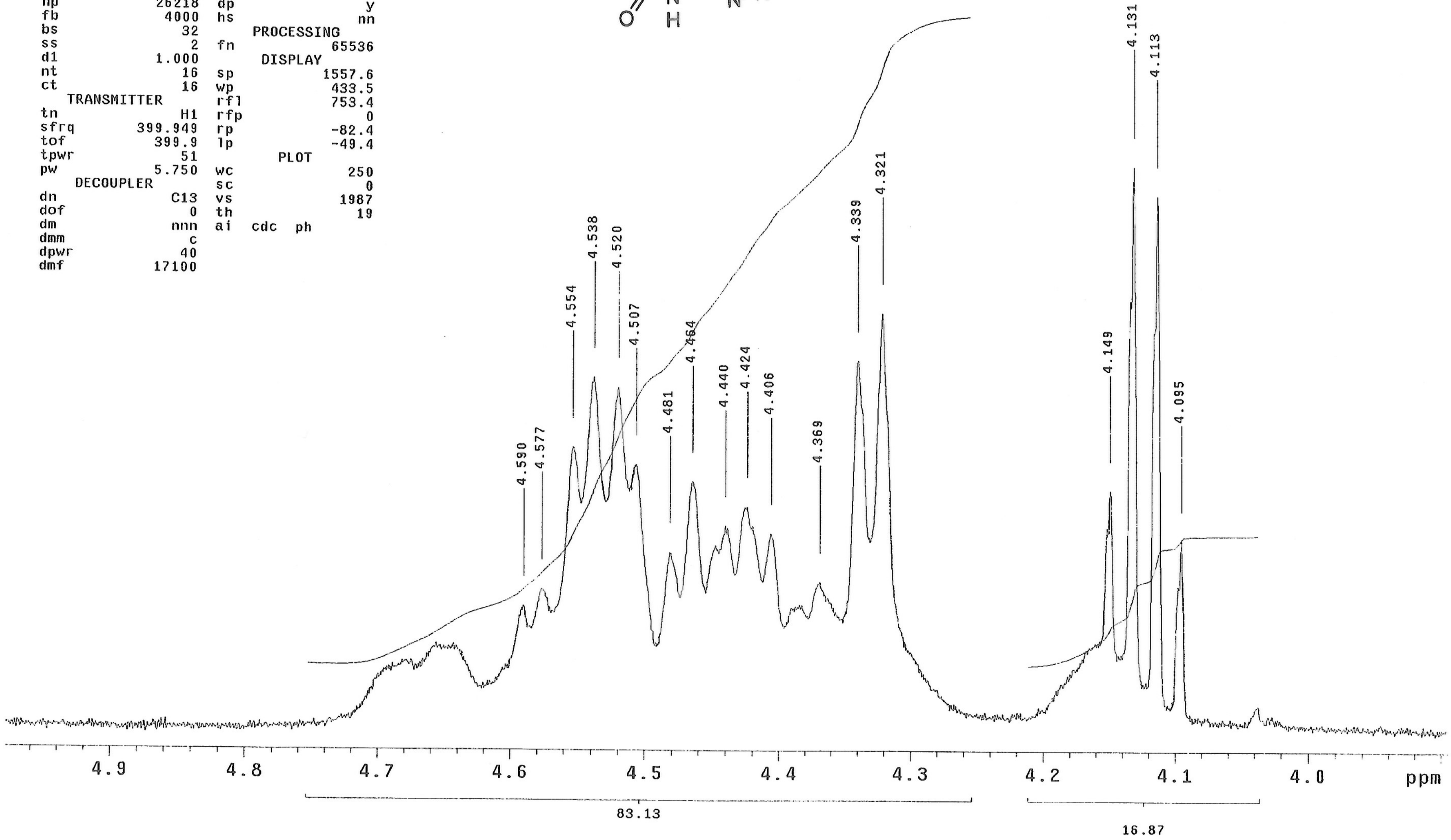

13-0203-011-25-1
col 1, fr 7 & 8

exp1 Proton

SAMPLE		SPECIAL	
date	Jul 7 2015	temp	24.0
solvent	cdcl3	gain	not used
file	/home/pharmd/~	spin	20
vnmrsys/data/auto_~		hst	0.008
2015.06.09/0093.fi~		pw90	11.500
	d	alfa	6.600
ACQUISITION		FLAGS	
sw	6399.0	il	n
at	2.049	in	n
np	26218	dp	y
fb	4000	hs	nn
bs	32	PROCESSING	
ss	2	fn	65536
d1	1.000	DISPLAY	
nt	16	sp	265.4
ct	16	wp	1258.4
TRANSMITTER		rfl	753.4
tn	H1	rfp	0
sfrq	399.949	rp	-82.4
tof	399.9	lp	-49.4
tpwr	51	PLOT	
pw	5.750	wc	250
DECOUPLER		sc	0
dn	C13	vs	243
dof	0	th	13
dm	nnn	ai cdc	ph
dmm	c		
dpwr	40		
dmf	17100		

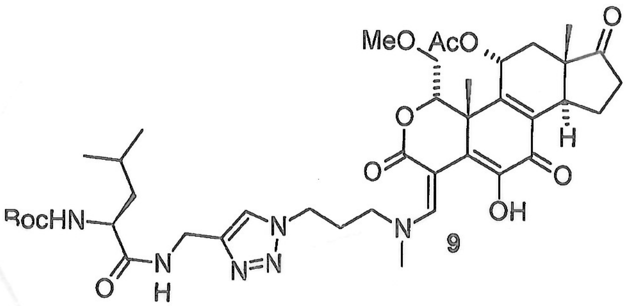

9

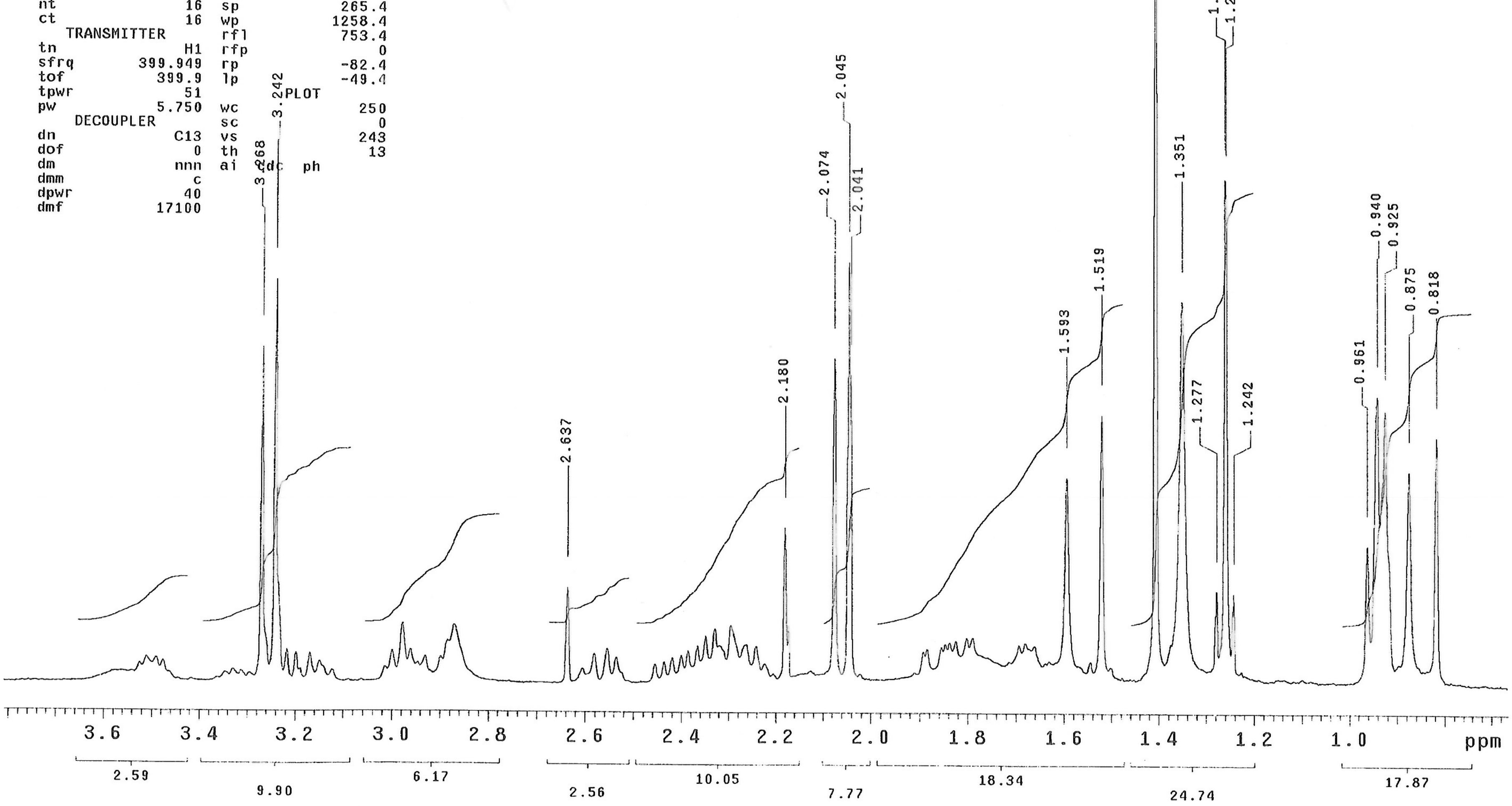

Print of window 80: MS Spectrum
13-0203-011-25-2
col 1, fr 7-8

==

Injection Date	: 7/7/2015 12:00:54 PM	Seq. Line :	1
Sample Name	: 130203011-25-2	Location :	Vial 14
A Operator	: Synthesis	Inj :	1
Acq. Instrument	: Instrument 1	Inj Volume :	5 µl
Method	: C:\HPCHEM\1\METHODS\GENPOS1.M		
Last changed	: 2/18/2015 9:32:10 AM by		
General use pos			

MS Spectrum

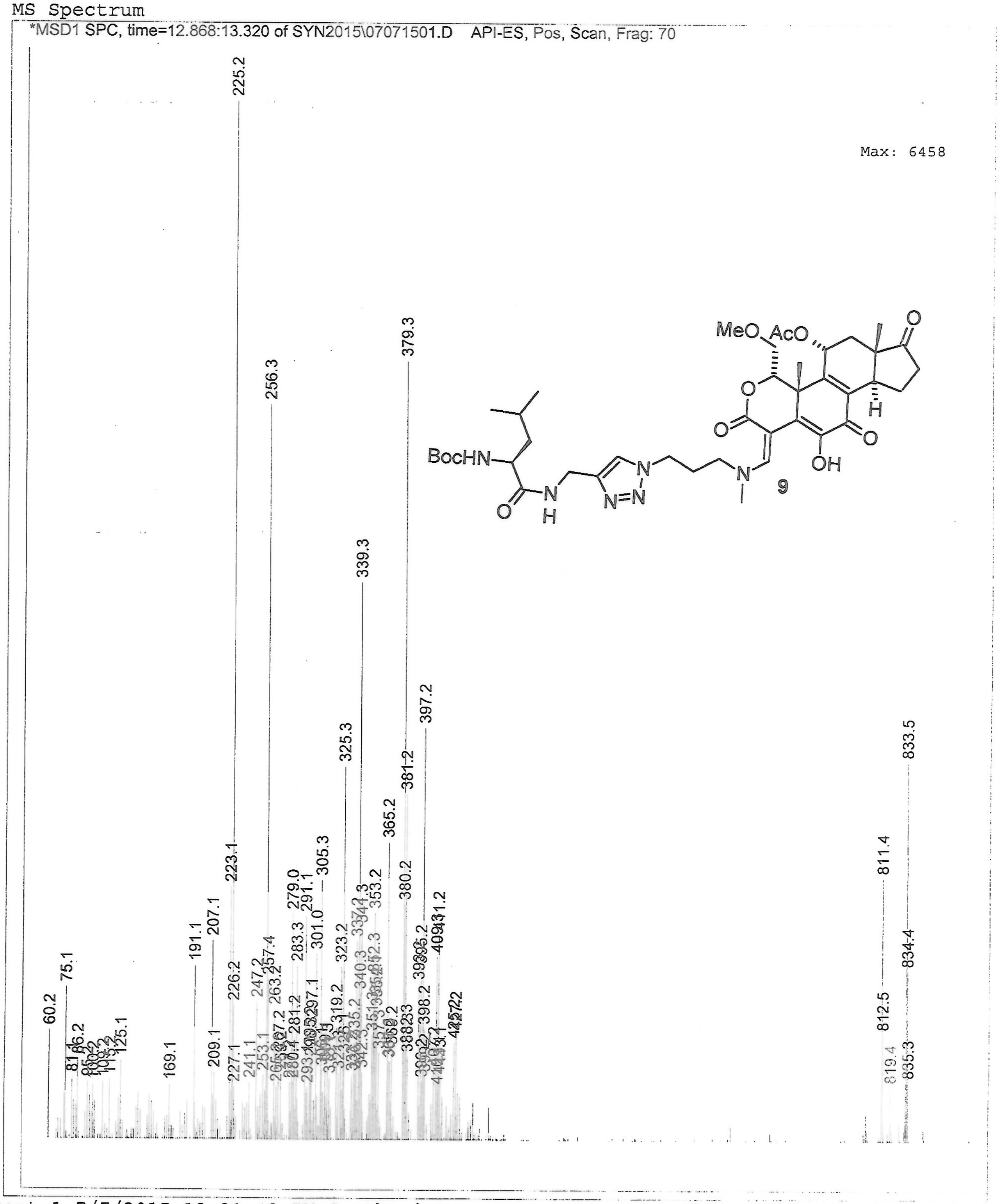

13-0203-011-26-35
W. [illegible] 07/16/15

Std proton
13-0203-011-26-1

exp2 Proton

SAMPLE		SPECIAL	
date	Jul 16 2015	temp	24.0
solvent	cdcl3	gain	not used
file	exp	spin	not used
ACQUISITION		hst	0.008
sw	6399.0	pw90	11.500
at	2.049	alfa	6.600
np	26218	FLAGS	
fb	4000	il	n
bs	32	in	n
ss	2	dp	y
d1	5.000	hs	nn
nt	64	PROCESSING	
ct	32	lb	0.50
TRANSMITTER		fn	65536
tn	H1	DISPLAY	
sfrq	399.949	sp	-785.2
tof	399.9	wp	6398.8
tpwr	51	rfl	785.4
pw	5.750	rfp	0
DECOUPLER		rp	-108.9
dn	C13	lp	-38.7
dof	0	PLOT	
dm	nnn	wc	250
dmm	c	sc	0
dpwr	40	vs	893
dmf	17100	th	3
		ai cdc ph	

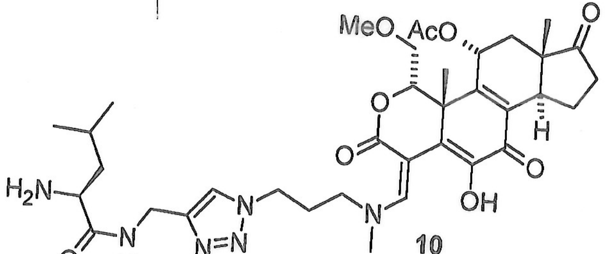

10

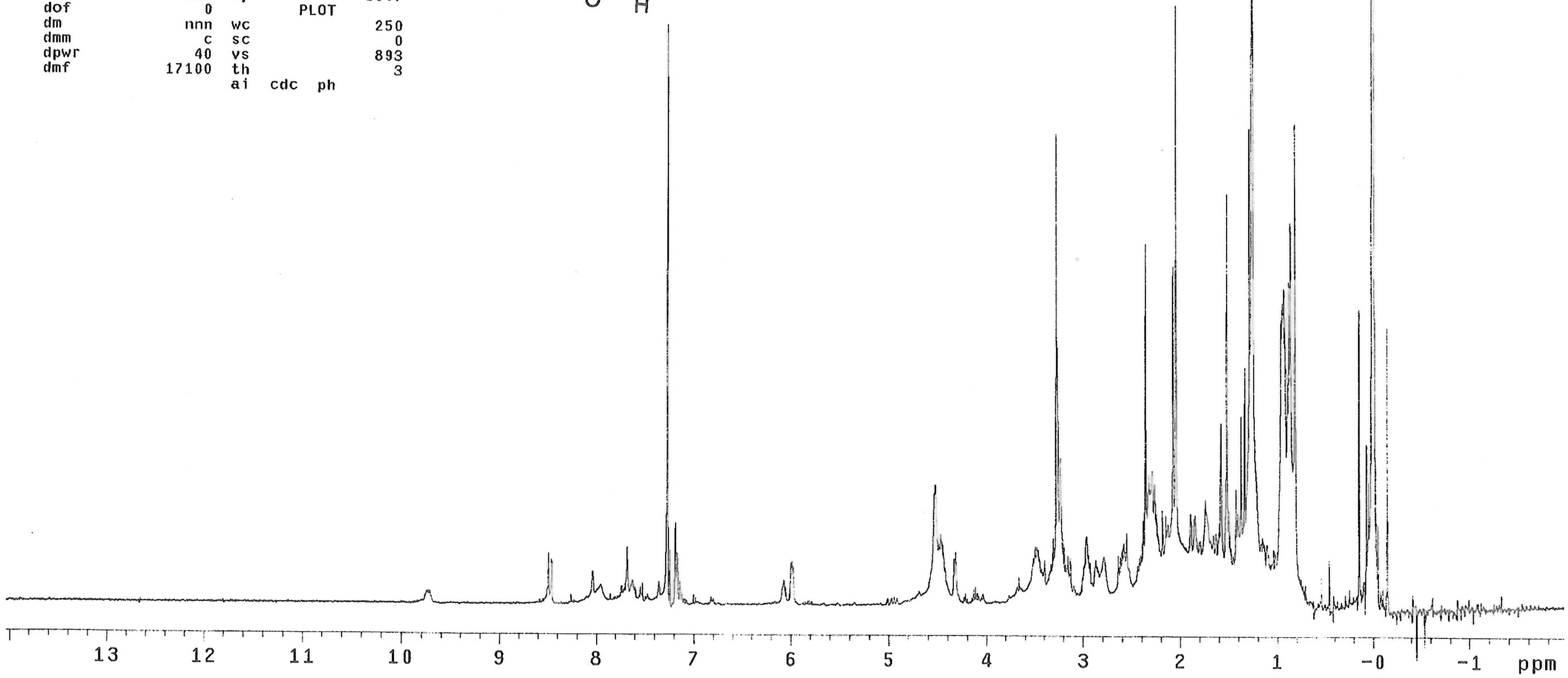

Print of window 80: MS Spectrum
13-0203-011-26-3
crude product

===
Injection Date : 7/16/2015 10:13:32 AM   Seq. Line : 1
Sample Name : 130203011-26-3   Location : Vial 17
[illegible] Operator : Synthesis   Inj : 1
Acq. Instrument : Instrument 1   Inj Volume : 5 μl
Acq. Method : C:\HPCHEM\1\METHODS\GENPOS1.M
Last changed : 2/18/2015 9:32:10 AM by
Analysis Method : C:\HPCHEM\1\METHODS\GENPOS2.M
Last changed : 6/26/2015 10:29:14 AM by Synthesis
General use pos

MS Spectrum

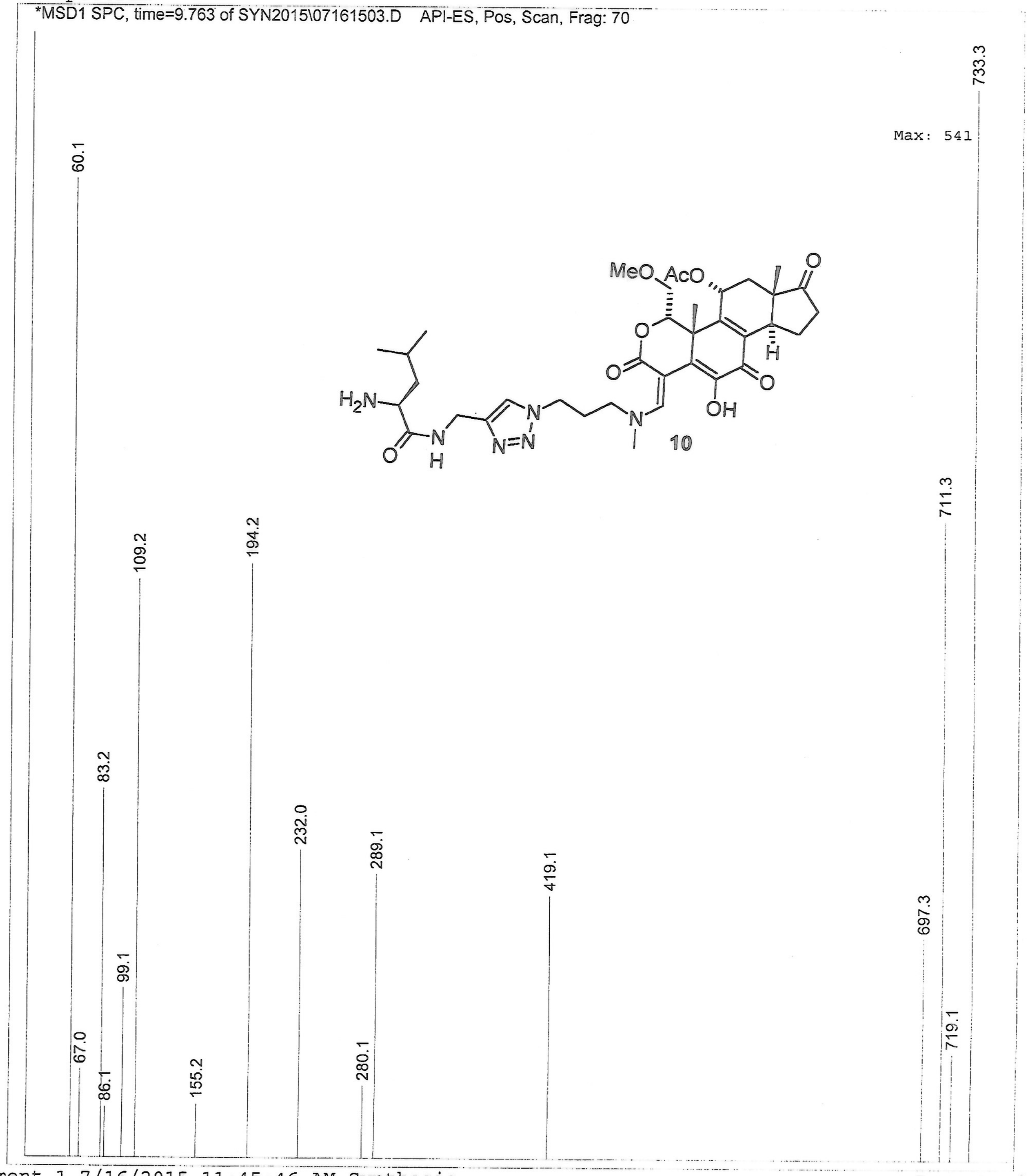

Supplement: Supplementary file 1 [file molecules-23-01791-s001.pdf]
